# Spatial association of seabirds and aquatic birds with highly pathogenic avian influenza (H5N1) outbreaks in Brazil: A nationwide ecological and statistical modelling approach

**DOI:** 10.1371/journal.pone.0350505

**Published:** 2026-07-01

**Authors:** Byron Abdel Hernández-Ortiz, Thiago Araujo dos Santos, Camila Michele Appolinário, Angela Maria Arcila-Cardona

**Affiliations:** 1 Department of Animal Production and Veterinary Preventive Medicine, School of Veterinary Medicine and Animal Science, São Paulo State University (UNESP), Botucatu, Sao Paulo, Brazil; 2 Colombian Corporation for Agricultural Research (AGROSAVIA), Cundinamarca, Colombia; National Veterinary Research Institute, NIGERIA

## Abstract

Identifying wild bird species associated with highly pathogenic avian influenza (HPAI) is essential for optimizing surveillance and mitigating spillover risks. This study analyzes Brazil’s nationwide HPAI surveillance data (up to July 2025), comprising 1,153 records across 127 bird and mammal species from 525 municipalities. Using a multi-model framework—including chi-square association tests, binary logistic regression, and spatial Generalized Additive Models (GAMs). Species–outbreak associations were significative and positive for *Thalasseus maximus* (χ² = 237.34, p < 0.0001), *Thalasseus acuflavidus* (χ² = 216.12, p < 0.0001), *Sterna hirundo* (χ² = 83.88, p < 0.0001), and *Sterna hirundinacea* (χ² = 77.56, p < 0.0001). An optimized logistic regression model (model 3) highlighted *T. acuflavidus* as the strongest predictor of HPAI (OR = 80.74; 95% CI: 21.85–298.39; p < 0.001), achieving good predictive performance (AUC = 0.85; Pseudo R² = 0.48). To account for spatial dependence, we fit a binomial GAM incorporating a bivariate longitude–latitude spatial smoother, significantly improving model fit (AUC = 0.96; Pseudo R² = 0.58) and effectively accounting for residual spatial autocorrelation (Moran’s I = 0.0399, z = 2.10, p = 0.044). Outbreaks were concentrated along Brazil’s southeastern coast, overlapping with high-density poultry zones, while inland spread remained sporadic, suggesting migratory routes as key transmission pathways. These results underscore the critical role of seabirds—particularly *T. acuflavidus*—in HPAI H5N1 dynamics in Brazil. The enhanced predictive power of the spatial GAM supports its utility in risk mapping. We recommend integrating biodiversity data with spatial modeling to guide targeted surveillance in high-risk coastal areas, reducing spillover threats to poultry and wild populations.

## Introduction

Highly pathogenic avian influenza (HPAI) poses a significant threat to the health of birds and mammals worldwide, primarily affecting wild, migratory, and domestic bird species. Its spread has caused extensive outbreaks with severe ecological and economic consequences. To date, the disease has been reported in approximately 108 countries across six continents, including Antarctica, resulting in the deaths of over 300 million birds globally [1,2]. The high mortality associated with HPAI is largely due to the virus’s ability to cross interspecies barriers, having infected more than 500 bird species and at least 70 mammal species, including vulnerable species such as the California condor (*Gymnogyps californianus*) and polar bears (*Ursus maritimus*) [1].

Recent HPAI outbreaks have raised concerns among health authorities as they pose risks to food safety, poultry production, and the stability of affected ecosystems [3]. The presence of the H5N1 virus in the Americas was first documented in wild birds in Canada in late 2021, followed by outbreaks in poultry in the United States in February 2022. Between 2022 and epidemiological week 8 of 2025, a total of 19 countries and territories in the Americas reported 4,713 H5N1 avian influenza outbreaks to the World Organization for Animal Health (WOAH), representing an increase of 325 outbreaks compared to the most recent epidemiological update published by the Pan American Health Organization (PAHO/WHO) on January 24, 2025 [4].

In Brazil, avian influenza has been documented since the 1970s, serological studies conducted in wild and domestic birds in São Paulo [5] identified the presence of the A/duck/England/56 (H11N6) virus. During the 1980s, in Rio de Janeiro, isolates of A/turkey/Massachusetts/65 (H6N2) and A/duck/England/63 (H1N4) subtypes were reported in wild duck (*Dendrocygna viduata*) droppings and in exotic bird cages [5]. The first case of HPAI (H5N1) in wild birds was reported in May 2023, followed by detections in backyard domestic birds in June of the same year in Espírito Santo [6]. On May 15th 2025, Brazil confirmed its first HPAI outbreak in commercial poultry—specifically, in a breeder farm in Montenegro, Rio Grande do Sul—representing the first detection in the commercial sector [7]. A contingency plan was immediately implemented, and Brazil began the 28-day standstill period required to regain its disease‑free status. By late June 2025, Brazil had completed decontamination of the affected facility and initiated the formal process to notify WOAH (formerly OIE) of its renewed HPAI‑free estatus, pending official recognition [8].

The ecological impact of HPAI is substantial, with reports of significant declines in seabird populations and potential cascading effects on local ecosystems. The loss of key species can disrupt fundamental ecological interactions, including competition, predation, and symbiosis, ultimately affecting biodiversity [9–11]. Additionally, ecological connectivity and inter-ecosystem flows amplify HPAI effects, as bird population declines can negatively impact other species that depend on them for food or as part of the trophic network [12,13].

HPAI transmission is a complex process, with aquatic and seabirds—particularly those from the orders *Anseriformes* and *Charadriiformes*—recognized as natural reservoirs of various viral subtypes [1]. However, the H5N1 strain has demonstrated an increasing ability to accumulate mutations that enhance its pathogenicity in wild populations. Recent research suggests that H5N1 transmission dynamics involve multiple pathways, extending beyond conventional interactions between wild and domestic aquatic birds. Ecological interconnectedness, driven by migratory patterns, further complicates virus monitoring and control, as infected birds can travel long distances and spread the pathogen across diverse habitats [14].

Challenges associated with avian influenza are exacerbated by environmental factors such as climate change and habitat degradation, which can alter bird migratory patterns and increase the risk of virus transmission to domestic populations [11]. In this context, conserving Brazilian ecosystems depends on integrated monitoring strategies and collaborative efforts to mitigate the multiple risk factors associated with HPAI.

Avian influenza surveillance in Brazil has been an interinstitutional effort. Given that the country is the world’s third-largest poultry meat producer and the leading exporter, the Ministry of Agriculture, Livestock, and Supply (MAPA) implemented the Avian Influenza and Newcastle Disease (ND) Surveillance Plan in July 2022 [15]. This plan aims to: (i) detect cases early in domestic and wild birds; (ii) demonstrate the absence of these diseases in industrial poultry farming, in accordance with international surveillance guidelines for trade; and (iii) monitor viral strain circulation to guide public health and animal health strategies [15].

One of the most significant outcomes of this program is the creation of databases and information systems that enable the management of avian influenza epidemiological surveillance. Official records are available in a government repository covering data from all 26 federal units of Brazil, with information collected since March 2021 (https://www.gov.br/agricultura/pt-br/assuntos/noticias/mapa-disponibiliza-painel-sobre-focos-confirmados-de-influenza-aviaria).

This study provides a comprehensive analysis of cases reported in the official repository up to July 7, 2025, focusing on the taxonomic composition of reports and the association between the most frequently affected species and the probability of HPAI outbreaks. To achieve this, we performed an association analysis between species with the highest number of records and the municipalities where they were detected, complemented by a binary logistic regression approach. The findings of this study offer critical insights into the ecology of virus–host interactions and support the optimization of epidemiological surveillance strategies by prioritizing high-risk areas, particularly those relevant to commercial poultry production. This can be achieved through targeted monitoring of wild bird abundance in species strongly associated with outbreak occurrence, providing a scientific basis for proactive risk mitigation and improved resource allocation.

## Materials and methods

### Data sources and surveillance context

The methodological framework and presentation of the results followed the RiGoR checklist proposed by Kerr et al. [16]. This checklist was used to improve transparency in the development of the risk models and to make explicit the main sources of potential bias that could affect the interpretation of the results.

The data analyzed in this study came from Brazil’s official surveillance system for avian influenza and Newcastle disease, coordinated by the Department of Animal Health of the Ministry of Agriculture and Livestock (DSA/MAPA) and implemented by state veterinary services. This surveillance system is designed primarily for early detection of suspected cases and outbreaks. It combines passive surveillance, based on the notification of suspected events in poultry and wild birds, with active and risk-based surveillance in selected poultry systems, backyard flocks, and certified compartments.

Passive surveillance records are generated when suspected respiratory or neurological disease in birds is reported. These reports may involve unusual mortality, compatible clinical signs, abnormal behavior in wild birds, epidemiological links with confirmed or suspected cases, or positive screening tests. In wild birds, unusual mortality events are considered situations in which dead or sick birds are found in numbers above those normally expected and without an evident alternative cause, excluding events clearly attributable to anthropogenic or natural causes. Notifications may be made by environmental agencies, producers, technical personnel, veterinarians, or any citizen, and are directed to the Official Veterinary Service at the local veterinary unit responsible for the municipality where the event is detected. Once notified, field investigations are conducted by the Official Veterinary Service and include site inspection, preferably with the support of an environmental professional, interviews with the person or institution that found or reported the birds, verification of abnormal mortality or compatible clinical signs, epidemiological investigation, sample collection, and registration of the event in e-Sisbravet.

All suspected events that met the official criteria for probable respiratory and neurological syndrome were submitted for sampling according to the passive surveillance protocols. Biological material included tracheal and cloacal swabs and, when necropsy was possible, tissues or organs from affected or recently dead birds without evidence of organ autolysis. Organ samples corresponded mainly to the digestive, respiratory, and nervous systems. Samples were first tested for molecular detection of influenza A. Positive or inconclusive samples were then tested for H5, H5 clade 2.3.4.4, and N1 by RT-qPCR. In selected cases, partial sequencing of the hemagglutinin gene was also performed [17].

Because these data come from an official surveillance system, they should not be interpreted as a random or fully representative sample of all bird species, all geographic areas, or all infection events in Brazil. The surveillance system is intentionally focused on early warning and risk-based detection. Therefore, it gives particular attention to aquatic and migratory birds, especially species from the orders Anseriformes and Charadriiformes, as well as to mortality events detected in coastal areas, wetlands, migratory routes, poultry-producing regions, and areas with stronger environmental monitoring capacity.

This surveillance design may lead to sampling imbalance. Species that are large, colonial, water-associated, abundant, or easier to detect are more likely to be reported and sampled. In contrast, subclinical infections, isolated deaths in remote areas, small or cryptic species, and taxa with low probability of human detection may be underrepresented. For this reason, the species-level associations and spatial patterns identified in this study were interpreted as signals emerging from surveillance data, rather than as direct estimates of infection prevalence in wild bird populations.

For the analysis, we used two national datasets from MAPA, updated to July 7, 2025. The first dataset, DB1, entitled *Collection of Suspected HPAI Samples in Brazil*, includes both suspected and confirmed events. It contains information on the federal unit and municipality, occurrence ID, date of veterinary service, main species recorded, farming or management type, case status, and date of conclusive diagnosis (the complete dataset is included as S1 File). The second dataset, DB2, entitled *HPAI Outbreak Data in Brazil*, is a complementary georeferenced register of confirmed HPAI and Newcastle disease events. It includes the disease diagnosis, case status, farming type, location, scientific name of the species, main-species indicator, investigation number linked to DB1, report date, and geographic coordinates (the complete dataset is included as S2 File).

Before analysis, the datasets were cleaned and harmonized. Records in DB1 in which the field “main species” referred to broad or mixed taxonomic categories, such as “duck” or “parrot,” were excluded because they could not be reliably assigned to a single species. Scientific names were standardized, and each species was classified into ecological groups, including seabirds, aquatic birds, terrestrial birds, domestic birds, and mammals. Migratory status for bird species was assigned following Somenzari et al. [18]. For DB2, geographic coordinates were checked to confirm that all georeferenced records were located within Brazilian territory.

### Outcome definition

The binary outcome was municipality-level outbreak status (≥1 confirmed HPAI outbreak = 1; none = 0), derived from DB1, which contains both suspected and confirmed events and the final case status per occurrence. For geospatial mapping and spatial diagnostics that require point locations, we used DB2 solely to obtain geographic coordinates of confirmed foci; DB2 does not alter the outcome definition, which remains anchored in DB1.

### Predictor variables and data filtering

We tallied species-specific records per federal unit and municipality. To ensure representativeness and model stability and reduce noise from rare species, we applied a preliminary filtering step to the original dataset. We included: (i) all species from the family Anatidae, given their well-documented role in the epidemiology of avian influenza viruses worldwide, and (ii) all species with 10 or more records in the database. This threshold reflected the right-skewed distribution of records per species (mean = 9; median = 2; interquartile range = 1–5; maximum = 421), where 75% of species had five or fewer records and only 12.5% exceeded ten records. This procedure resulted in a subset of 23 species, which were considered as potential predictors for subsequent modeling steps.

### Statistical analysis

We summarized cases by federal unit and municipality and computed outbreak frequency by species. Associations between the four species with the highest HPAI-positive proportions and municipal outbreaks were tested using χ². We then fit three models of increasing complexity:

#### Model 1: Total records as predictor.

As a baseline approach, we constructed a logistic binary regression model using the total number of records per municipality as the sole predictor. Analysis was performed using the Statsmodels module in Python **[19]**. This model served to evaluate the association between sampling intensity and the occurrence of HPAI outbreaks.

#### Model 2: LASSO-based selection from 23 species.

The second model aimed to identify the most relevant species associated with HPAI occurrence from the filtered set of 23 species. We applied the Least Absolute Shrinkage and Selection Operator (LASSO) regularization technique within a logistic regression framework. LASSO introduces an L1 penalty on the magnitude of coefficients, forcing non-informative predictors toward zero and effectively performing variable selection [20]. The penalty parameter (λ) was tuned using five-fold cross-validation, optimizing for predictive performance measured by the area under the ROC curve (AUC). This method is particularly suitable for high-dimensional ecological data with potential multicollinearity among predictors. Species with non-zero coefficients after penalization were retained.

#### Model 3: Optimized model.

From the set of species selected by LASSO, we built an optimized logistic regression model including only those species that remained statistically significant (p < 0.05) after multivariable adjustment. This final step ensured interpretability and parsimony by excluding species with unstable or marginal effects.

#### Model evaluation.

For each model, we reported estimated coefficients, odds ratios (OR) with 95% confidence intervals (CI), and p-values. Model fit was assessed using McFadden’s Pseudo R². Predictive performance was evaluated by constructing Receiver Operating Characteristic (ROC) curves and calculating the Area Under the Curve (AUC). All analyses were performed in Python using the Statsmodels [19] and Scikit-Learn [21] libraries.

To minimize resubstitution and model selection bias, all models were developed following a predefined three-step analytical framework (baseline, penalized, and optimized). The penalty parameter (λ) for the LASSO model was tuned using five-fold cross-validation, and predictive performance (ROC and AUC) was assessed through cross-validation folds rather than the training data. This sequential design ensured that performance estimates were unbiased and that all intermediate results were transparently reported.

#### Assessment of spatial autocorrelation.

To evaluate the spatial independence of the residuals from the optimized logistic regression model, we applied Moran’s I test [22] using a k-nearest neighbors (kNN, Haversine) spatial weights matrix. Geographic coordinates for each municipality were obtained from the centroid of its administrative polygon (EPSG:4326) and used to construct the spatial weights (the complete data set is included as S3 File).

The kNN approach was implemented with k = 8 neighbors per observation, ensuring a balanced representation of local spatial relationships while avoiding disconnected observations. The weights were row-standardized to ensure comparability across municipalities. Raw residuals were extracted from the optimized logistic regression model and tested for spatial autocorrelation using 9,999 random permutations to assess significance.

The Moran’s I statistic, z-score, and p-value were computed using the PySAL 2.8.0 and ESDA Python libraries [23], and the results were interpreted under a two-tailed significance framework (α = 0.05).

To account for potential spatial autocorrelation in the residuals of the optimized logistic regression model, a Generalized Additive Model (GAM) [24], with an explicit spatial term was implemented. The binomial GAM combined a bivariate spatial smooth over municipality centroids (longitude, latitude) with the predictors from the optimized model specified as linear terms, a decision based on interpretability, data sparsity for some species, and model parsimony. Smoothness parameters were selected by generalized cross-validation (GCV). Spatial independence of the GAM residuals was reassessed using Moran’s I and Local Indicators of Spatial Association (LISA), with significance determined via 9,999 Monte Carlo permutations.

#### Geospatial mapping and hotspot analysis.

We mapped outbreaks and species distributions using ArcMap 10.5 **[25]**. These maps integrated the following layers: i. Federal administrative boundaries (Source: Brazilian Institute of Geography and Statistics – IBGE, State Mesh (2022)), ii. Locations of confirmed HPAI outbreaks recorded in the official repository (cut-off date: July 2025), and iii. Geographic distribution of bird species with the highest proportion of HPAI-positive records.

The distribution of the chosen species was generated using the information available at the Global Biodiversity Information Facility (GBIF) database (https://www.gbif.org/). To mitigate sampling bias and facilitate statistical analysis, point data were aggregated into areal units. A grid of hexagonal polygons was chosen over squares for its superior ability to represent spatial continuity and reduce directional bias [26]. The hexagons were generated at a resolution of 10000 km² using the Generate Tessellation tool in ArcGIS 10.5 [25]. The number of observation points within each hexagon was summarized using the Spatial Join tool, resulting in a new field, Join_Count, which served as the input value for the subsequent hotspot analysis.

Spatial clustering of high-value observations (hotspots) and low-value observations (coldspots) were identified using the Getis-Ord Gi statistic* [27], a local spatial autocorrelation measure implemented in the Hot Spot Analysis (Getis-Ord Gi*) tool (ArcGIS Spatial Statistics Toolbox).

The analysis was performed on the hexagonal grid layer with the Join_Count as the input field. Spatial relationships were conceptualized using an Inverse Distance model, which assigns a higher weight to nearby features than to distant ones, based on the ecological premise that the influence of observations decays with distance [28]. The Euclidean distance method was used to calculate the distance between polygon centroids. A fixed distance band was not specified; instead, the software’s default threshold was applied, which ensures every feature has at least one neighbor. This approach was selected over contiguity-based methods (e.g., Queen’s Case) to more accurately model continuous ecological processes and to avoid edge-effect biases inherent in adjacency-based models for raster-like grids.

The tool outputs a new z-score and p-value for each feature. Features with a high positive z-score and a statistically significant small p-value (p < 0.05) indicate a spatial clustering of high values (“hotspots”). Conversely, features with a low negative z-score and a significant p-value indicate a spatial clustering of low values (“coldspots”). The results were visualized by symbolizing the output GiBin field, which classifies features into confidence intervals (e.g., 99% confidence hotspot, 95% confidence coldspot).

All spatial analyses were conducted within a projected coordinate system (SIRGAS 2000 Brazil Polyconic EPSG: 5880) to ensure accurate distance calculations. The final map was presented with the grid outlines removed to emphasize the spatial pattern of the hotspots.

### Ethics

This study used aggregated, publicly available surveillance and biodiversity data; no human or animal subjects were directly involved. Institutional review board approval was not required.

## Results

### Taxonomic composition of the database

**Fig 1:**
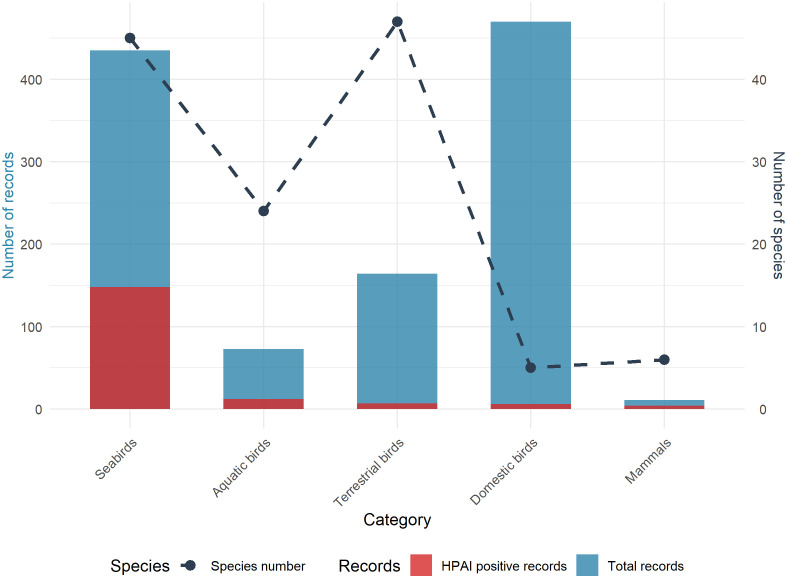
Species richness, sampling effort, and HPAI detections across avian and mammalian groups.

**Fig 2:**
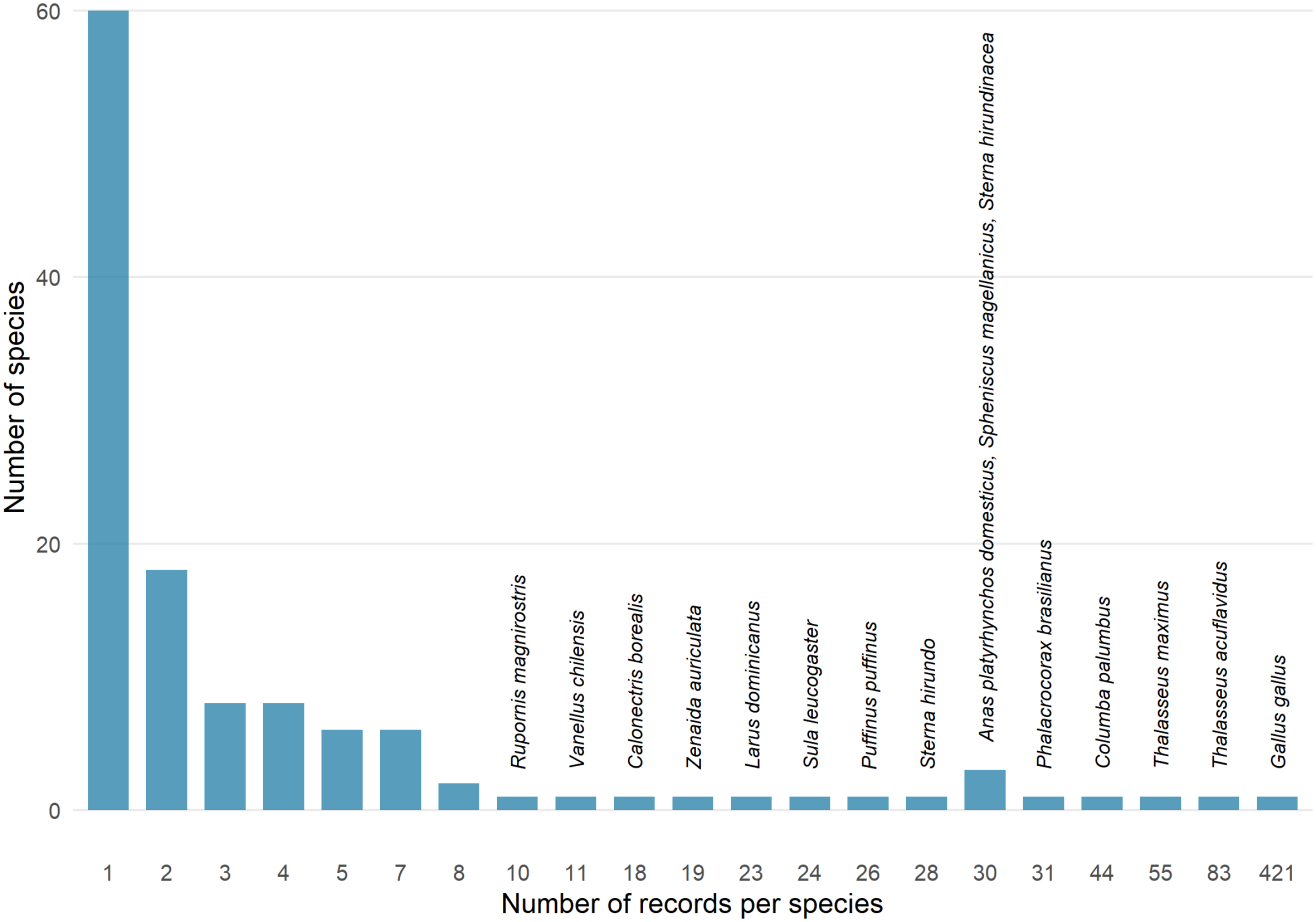
Sampling frequency distribution of species under HPAI surveillance in Brazil (as of July 2025).

The curated database (DB1) included 1,153 records from 127 animal species across 26 federal units and 525 municipalities, distributed as follows: 45 seabird species (of which 28 were migratory or partially migratory), 24 species of aquatic birds, 47 species of terrestrial birds, 5 domestic bird species, and 6 mammal species (Fig 1). Birds comprised 99% of the records, with the remaining 0.95% corresponding to marine, aquatic, and terrestrial mammals. The most frequently sampled avian species were *Gallus gallus* (421 records), *Thalasseus acuflavidus* (83), *Thalasseus maximus* (55), *Columba palumbus* (44), *Phalacrocorax brasilianus* (31), *Anas platyrhynchos domesticus* (30), *Sterna hirundinacea* (30), *Spheniscus magellanicus* (30), *Sterna hirundo* (28), and *Puffinus puffinus* (26) (Fig 2).

Distribution of total species number, number of recorded samples (sampling effort), and confirmed HPAI-positive records across five avian ecological groups (seabirds, aquatic birds, terrestrial birds, domestic birds) and mammals. Seabirds show the highest species richness and number of total records, as well as the highest absolute number of HPAI-positive events, followed by aquatic and terrestrial birds. Domestic birds and mammals have comparatively fewer total species and records, with very low or single HPAI detections. This comparison highlights that high sampling effort and species richness do not always proportionally translate into HPAI positivity rates across groups.

The figure shows the number of species sampled with a given number of records (samples tested for suspected HPAI infection) in Brazil. Most species (60) were sampled only once, while 18 species were sampled twice, and eight species were sampled three or four times. A long tail of species with higher sampling effort is observed, culminating in the domestic chicken (*Gallus gallus*), which was sampled 421 times — far more than any other species. Other notably sampled species include *Thalasseus acuflavidus* (83 records), *Thalasseus maximus* (55 records), *Columba palumbus* (44 records), and several seabirds and aquatic birds with between 10 and 30 records. This distribution reveals that intensive sampling is concentrated in very few species, while most species contribute minimally to surveillance effort.

Although chickens (*G. gallus*) were the most sampled group, only four HPAI outbreaks were detected in subsistence-level domestic flocks and one in a commercial farm. In contrast, species from the Anatidae family (ducks, swans, and geese) accounted for nine positive outbreaks, with *D. viduata* and *Cygnus melancoryphus* each associated with three outbreaks. Among seabirds, positive HPAI cases were identified in 94.5% of *T. maximus* samples, 72.3% of *T. acuflavidus*, 42.9% of *S. hirundo*, and 26.7% of *S. hirundinacea*.

### Distribution of records by federal unit and municipality

**Fig 3:**
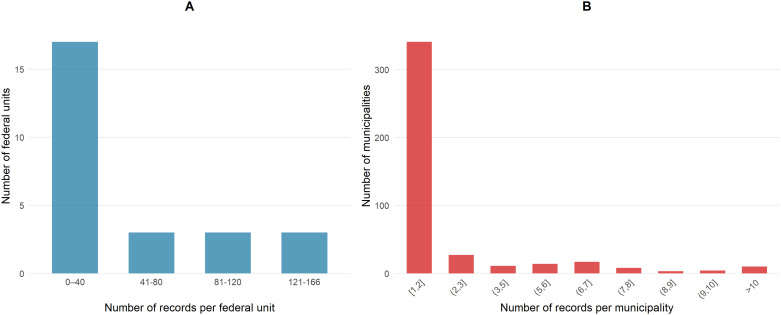
Distribution of HPAI suspect samples across Brazilian federal units and municipalities (as of July 2025). The figure presents the frequency distribution of HPAI suspect samples across two administrative levels in Brazil. (A) Brazilian federal units (states) are grouped into intervals based on the number of records. Seventeen federal units have between 0–40 records, three have 41–80, three have 81–120, and three have 121–166 records. This shows that most federal units contributed relatively few samples, while a small number of states concentrated the highest sampling effort. (B) At the municipal level, most municipalities contributed very few samples: most appear only once in the dataset. A long tail of municipalities with higher sampling includes Florianópolis (SC) with 23 records, Belo Horizonte (MG) with 18 records, Vila Velha (ES) with 17 records, São Sebastião (SP) with 15 records, Ubatuba (SP) with 14 records, Ilha Comprida (SP) and São Francisco do Sul (SC) with 13 records each, and several others (Rio de Janeiro, Santos, Itanhaém, Praia Grande, Penha, etc.) with 10–11 records. This distribution reveals that HPAI surveillance sampling in Brazil is highly concentrated in a few municipalities, while most municipalities contributed minimally to the total number of suspect samples.

Of the 1,153 records analyzed, 976 tested negative while 177 were confirmed HPAI outbreaks, yielding a national positivity rate of 15.3%. Sampling was focused on suspected cases, resulting in a heterogeneous distribution across federal units (UFs), ranging from a single record in Paraíba to 166 in Santa Catarina (Fig 3A). At the municipal level, 480 municipalities reported 1–5 samples, 31 reported 6–9, and only 16 reported ≥10 samples (Fig 3B).

**Fig 4:**
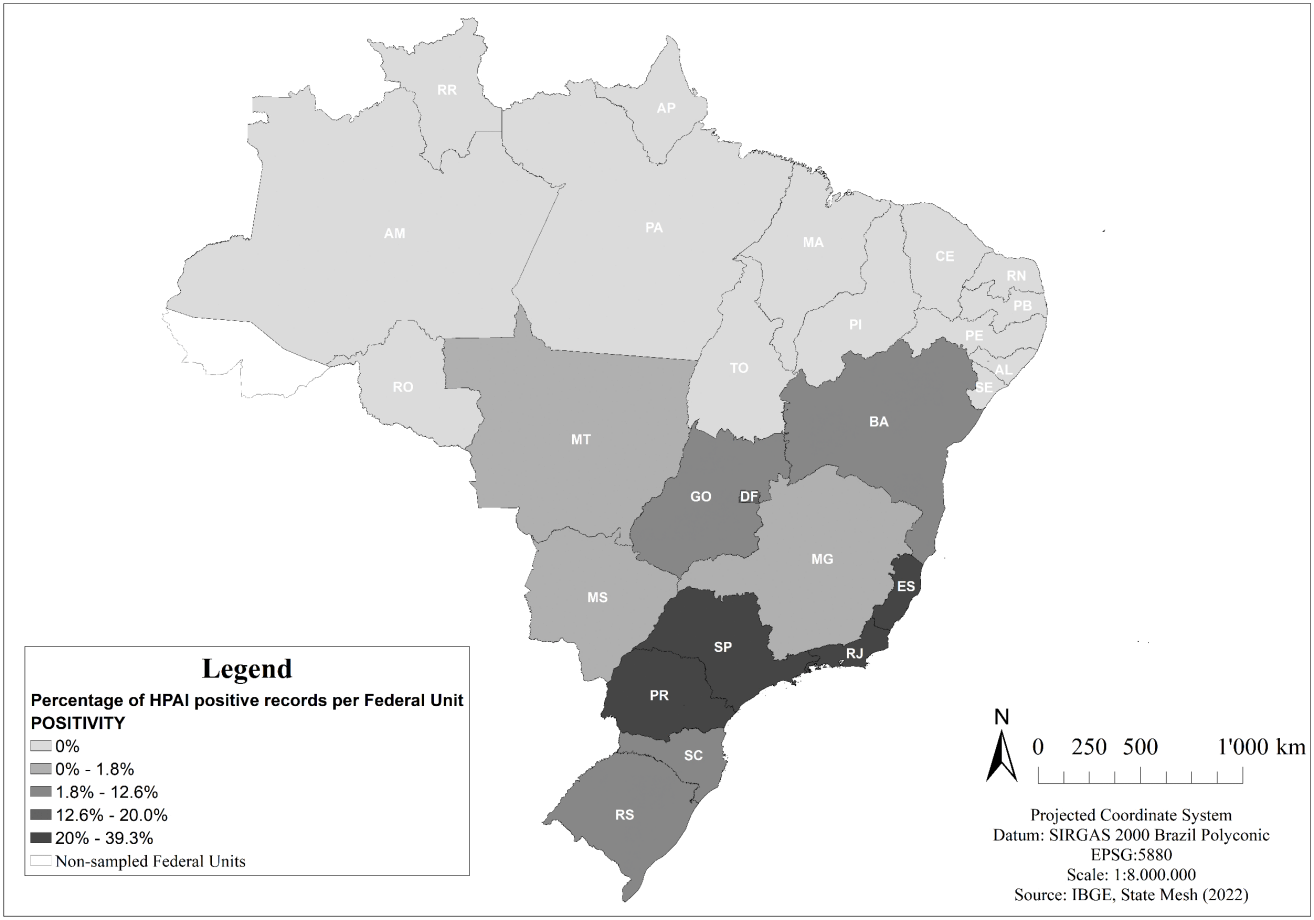
HPAI positivity rate per Brazilian federal unit (as of July 2025).

The average percentage of positive samples per UF was 8.23% ± 13.33. Fourteen UFs reported no outbreaks during the study period. The highest proportions of confirmed outbreaks were observed in São Paulo-SP (39.3%), Rio de Janeiro-RJ (37.3%), Espírito Santo-ES (37%), and Paraná-PR (34.2%), while the remaining UFs reported values below 20% (Fig 4)

Choropleth map showing the percentage of HPAI‑positive samples out of all suspect samples tested in each Brazilian state (federal unit). Positivity rates vary markedly across the country, with the highest proportions observed in São Paulo (39.31%), Rio de Janeiro (37.35%), Espírito Santo (37.00%), and Paraná (34.21%). Non‑sampled federal units are also indicated. Figure credit: Brazilian Institute of Geography and Statistics – IBGE, State Mesh (2022).

**Fig 5:**
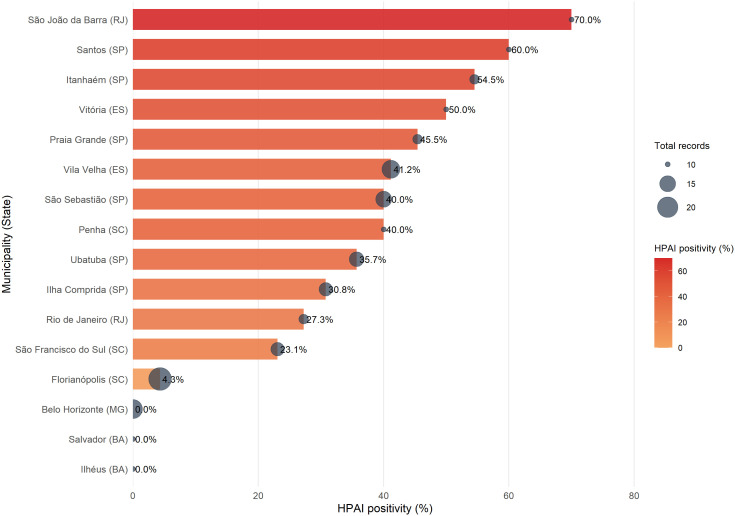
HPAI positivity rates in Brazilian municipalities with the highest sampling effort (≥10 suspect records, as of July 2025).

At the municipal level, the average proportion of positive samples was 7.53% ± 20.9. No outbreaks were detected in 444 municipalities, while the remaining 81 exhibited high variability, with positivity ranging from 4.3% to 100%. The proportion of positive cases differed markedly between municipalities with 1–9 records (13.4%) and those with ≥10 records (81.3%) (Fig 5). This pattern indicates a clustered sampling approach centered around detected outbreak sites

This figure shows the proportion of HPAI-positive samples among municipalities with ten or more suspect records in Brazil. Bars represent the positivity percentage, while point size is proportional to the total number of records per municipality. The highest positivity rates are observed in São João da Barra (RJ) with 70% positivity (10 records), Santos (SP) with 60% (10 records), Itanhaém (SP) with 54.5% (11 records), Vitória (ES) with 50% (10 records), and Praia Grande (SP) with 45.5% (11 records). Other municipalities with moderately high positivity include Vila Velha (ES: 41.2%, 17 records), Penha (SC: 40%, 10 records), São Sebastião (SP: 40%, 15 records), Ubatuba (SP: 35.7%, 14 records), Ilha Comprida (SP: 30.8%, 13 records), Rio de Janeiro (RJ: 27.3%, 11 records), and São Francisco do Sul (SC: 23.1%, 13 records). In contrast, Florianópolis (SC) shows a low positivity rate of 4.3% despite having the highest sampling effort (23 records). Three municipalities with ten or more records Ilhéus (BA), Salvador (BA), and Belo Horizonte (MG) recorded zero HPAI positivity. This figure demonstrates that high sampling effort does not always predict high positivity, revealing important local differences in HPAI detection across Brazil’s coastal and inland municipalities.

### Association analysis and binary logistic regression models

A significant association was found between the presence of specific bird species and the occurrence of HPAI outbreaks at the municipal level. The strongest associations were observed for: *T. maximus* (χ² = 237.34, p < 0.0001), *T. acuflavidus* (χ² = 216.12, p < 0.0001), *S. hirundo* (χ² = 83.88, p < 0.0001), and *S. hirundinacea* (χ² = 77.56, p < 0.0001).

After filtering the initial dataset to retain all Anatidae and all species with ≥10 records—a threshold reflecting the right-skewed distribution of records per species—a total of 23 species were included for modeling. Three logistic regression models of increasing complexity were developed and compared.

#### Model 1: Total records as a predictor.

This baseline model, using the total number of records per municipality as the sole predictor, demonstrated strong discriminative ability (AUC = 0.87) and moderate explanatory power (Pseudo R² = 0.331). Each additional record was associated with a 95% increase in the odds of an outbreak (OR = 1.94; 95% CI: 1.67–2.21; p < 0.001).

#### Model 2: LASSO-based selection.

The penalized regression approach selected 11 species as relevant predictors and achieved the highest predictive performance (AUC = 0.95; Pseudo R² = 0.74). However, several species exhibited inflated coefficients, suggesting quasi-complete separation and potential overfitting, which limited the interpretability of this model.

#### Model 3: Optimized model.

**Fig 6:**
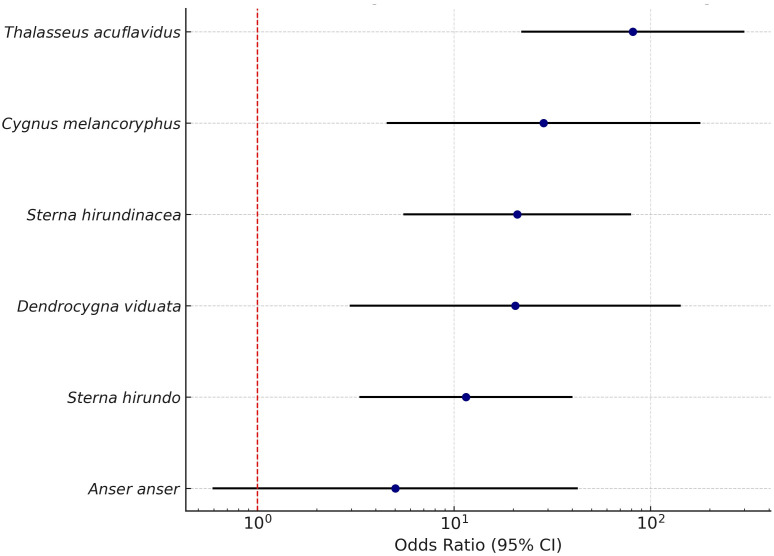
Seabird and aquatic bird species as strong predictors of HPAI occurrence: Odds ratios from a logistic regression model.

This model retained only the 6 species that remained statistically significant (p < 0.05) after multivariable adjustment, providing an effective balance between interpretability and predictive capacity (AUC = 0.85; Pseudo R² = 0.48). The strongest associations were observed among seabirds *T. acuflavidus* (OR = 80.74; 95% CI: 21.85–298.39; p < 0.001), *S. hirundinacea* (OR = 20.86; 95% CI: 5.49–79.2; p < 0.001), and *S. hirundo* (OR = 11.45; 95% CI: 3.28–39.95; p < 0.001). Significant associations were also detected for waterfowl *C. melancoryphus* (OR = 28.37; 95% CI: 4.51–178.31; p < 0.001) and *D. viduata* (OR = 20.39; 95% CI: 2.93–141.85; p = 0.002), whereas *Anser anser* did not reach statistical significance (p = 0.14) (Fig 6).

This forest plot displays odds ratios (OR) with 95% confidence intervals (CI) for six avian species included in the optimized logistic regression model predicting highly pathogenic avian influenza (HPAI) occurrence. The plot uses a logarithmic scale, with a vertical reference line at OR = 1 (no association). Species are ordered by effect size. The presence of *Thalasseus acuflavidus* shows the strongest association with HPAI occurrence (OR ≈ 100), followed by *Anser anser* (OR = 30), *Sterna hirundo* (OR = 25), *Dendrocygna viduata* (OR = 20), *Sterna hirundinacea* (OR = 18), and *Cygnus melanocoryphus* (OR = 15). All ORs are substantially greater than 1, and the confidence intervals (where available, e.g., *T. acuflavidus*: 95% CI not crossing 1) indicate statistical significance (p < 0.001 for top predictors). These results highlight that the presence of certain seabird and waterfowl species, particularly *T. acuflavidus*, is a strong risk factor or indicator for HPAI detection in Brazil’s surveillance system.

**Fig 7:**
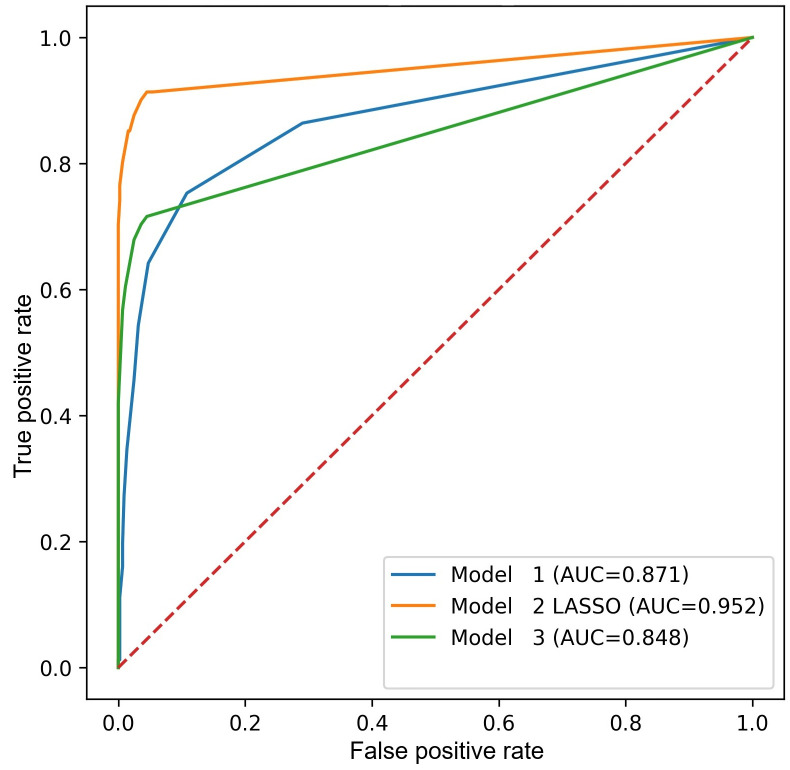
Receiver Operating Characteristic (ROC) curves comparing predictive performance of three HPAI occurrence models.

Comparative ROC curves (Fig 7) underscored the superior discriminative ability of the LASSO model. For practical and inferential purposes, the optimized model (Model 3) was selected as the most appropriate, as it combines robust predictive performance with biological plausibility.

This figure presents ROC curves for three logistic regression models predicting HPAI occurrence in Brazil, along with their respective Area Under the Curve (AUC) values. Model 1 (Total Records) shows moderate discriminative ability (AUC = 0.871). Model 2 (LASSO-Reduced) achieves the highest predictive performance (AUC = 0.952), indicating excellent discrimination between positive and negative cases. Model 3 (Optimized) has an AUC of 0.848, slightly lower than Model 1. The curves plot the true positive rate (sensitivity) against the false positive rate (1-specificity) across various classification thresholds. Model 2 consistently outperforms the other two models at all false positive rate levels below approximately 0.20, after which all models approach perfect sensitivity. These results suggest that the LASSO-based variable selection (Model 2) provides the best predictive accuracy, while Model 3 offers a compromise between performance and interpretability, albeit with a slight reduction in AUC compared to Model 1.

#### Spatial autocorrelation and generalized additive model (GAM).

Moran’s I applied to the raw residuals of the optimized logistic regression model indicated a statistically significant but low-magnitude positive spatial autocorrelation (I = 0.1078, z = 5.61, p = 0.002). This suggested that the model captured most of the spatial structure, but some residual dependence persisted, likely due to unmeasured environmental or ecological factors.

**Fig 8:**
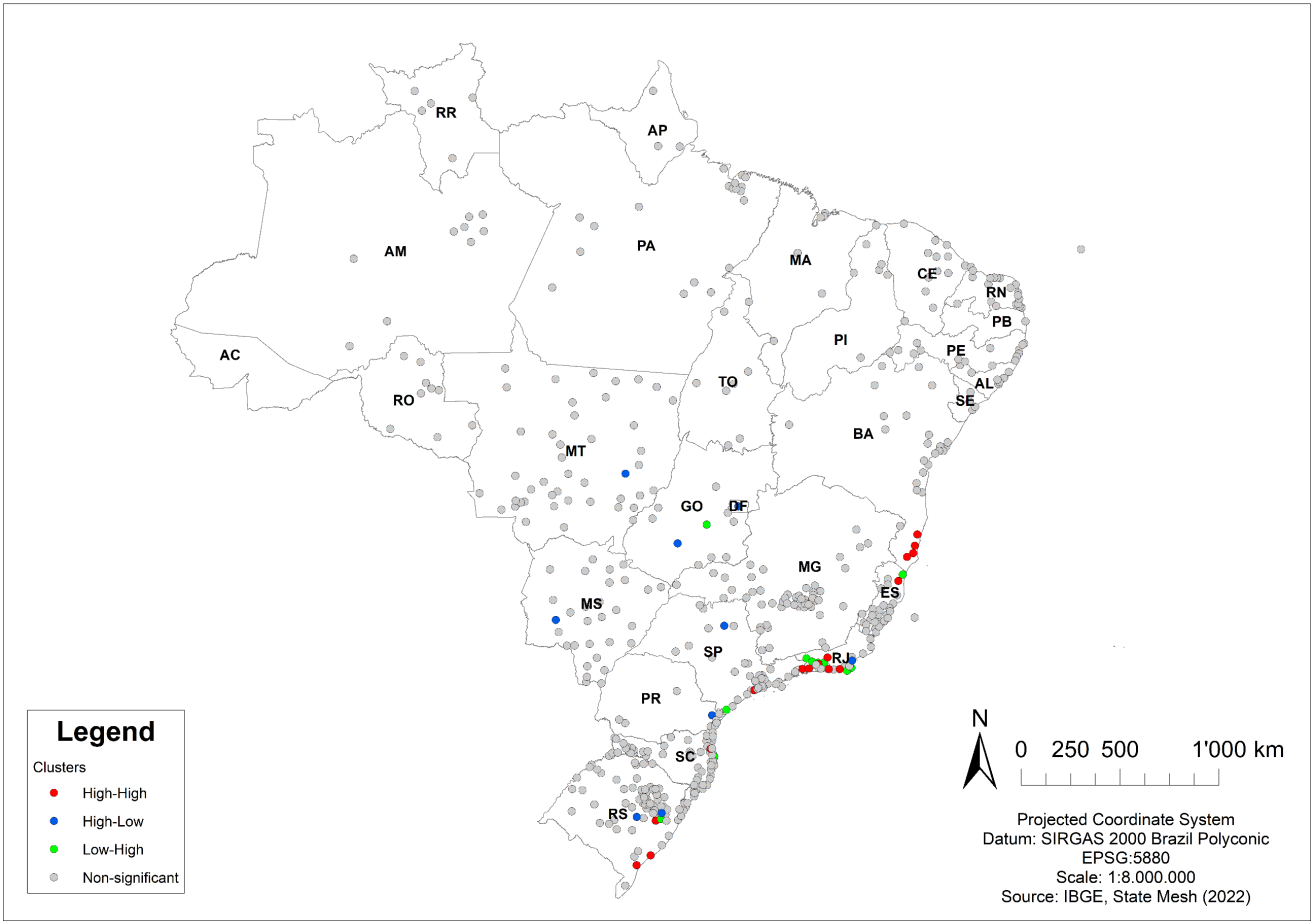
Spatial clustering analysis of HPAI model residuals across Brazilian federal units.

To address this, we implemented a spatial GAM incorporating a bivariate smooth of municipality centroids alongside the six species predictors as linear terms. The inclusion of the spatial smooth substantially improved model performance, increasing the AUC to 0.96 and the McFadden’s pseudo R² to 0.58. A subsequent Moran’s I test on the GAM residuals showed a marked reduction in spatial autocorrelation (I = 0.0399, z = 2.10, p = 0.044), indicating that the majority of the spatial pattern was successfully accounted for, though a minimal, yet detectable, structure persisted. LISA analysis of the GAM residuals revealed no consistent clustering, with most municipalities (92.8%) classified as non-significant and only small, spatially scattered clusters lacking coherent biological structure (Fig 8).

**Fig 9:**
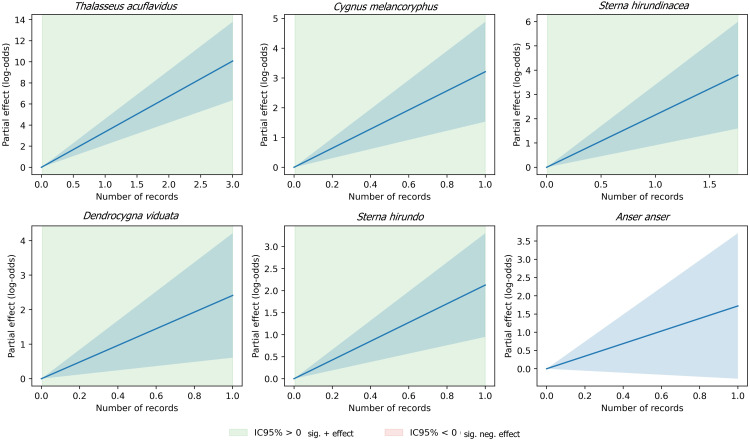
Partial effects of species occurrence on HPAI prediction from Spatial GAM.

Among the six predictor species in the spatial GAM, five showed statistically significant effects (p < 0.05): *T. acuflavidus* (p < 0.001), *C. melancoryphus* (p = 0.003), *S. hirundinacea* (p = 0.010), *S. hirundo* (p = 0.013), and *D. viduata* (p = 0.029). *Anser anser* was not significant (p = 0.089). The partial effects (Fig 9) revealed varying shapes: *T. acuflavidus* and *S. hirundinacea* displayed a positive, approximately linear association with HPAI risk, while *D. viduata* exhibited a moderate effect that plateaued at higher abundances, suggesting a saturation threshold.

Map presenting the results of a Local Indicators of Spatial Association (LISA) analysis applied to the raw residuals of a Spatial Generalized Additive Model (GAM) for HPAI occurrence in Brazil. Colored clusters indicate different types of spatial association: High‑High (HH) clusters, High‑Low (HL) outliers, Low‑High (LH) outliers, and non‑significant areas. Only a few small and scattered clusters are detected, with no clear or consistent spatial pattern of residual autocorrelation. Federal unit abbreviations follow IBGE standards. Figure credit: Brazilian Institute of Geography and Statistics – IBGE, State Mesh (2022).

This figure shows the estimated partial effects (log-odds) of key predictor species on HPAI occurrence derived from a Spatial Generalized Additive Model (GAM). The solid line represents the estimated partial effect as a function of the number of records for each species, while the shaded band indicates the 95% confidence interval (CI). Background shading highlights regions where the effect is significantly different from zero: positive if the 95% CI lies entirely above zero, negative if entirely below zero. For *Thalasseus acuflavidus*, the partial effect increases steeply and positively with the number of records, and the confidence interval remains above zero across most of the range, indicating a strong, statistically significant positive association with HPAI occurrence. For *Cygnus melancoryphus* (black-necked swan), preliminary data suggest a weaker or different pattern, though full interpretation requires complete data across the entire range of record counts. These partial effect plots allow visualization of the nonlinear relationships between species detection frequency and HPAI risk, after accounting for spatial autocorrelation in the model.

### Geospatial analysis

**Fig 10:**
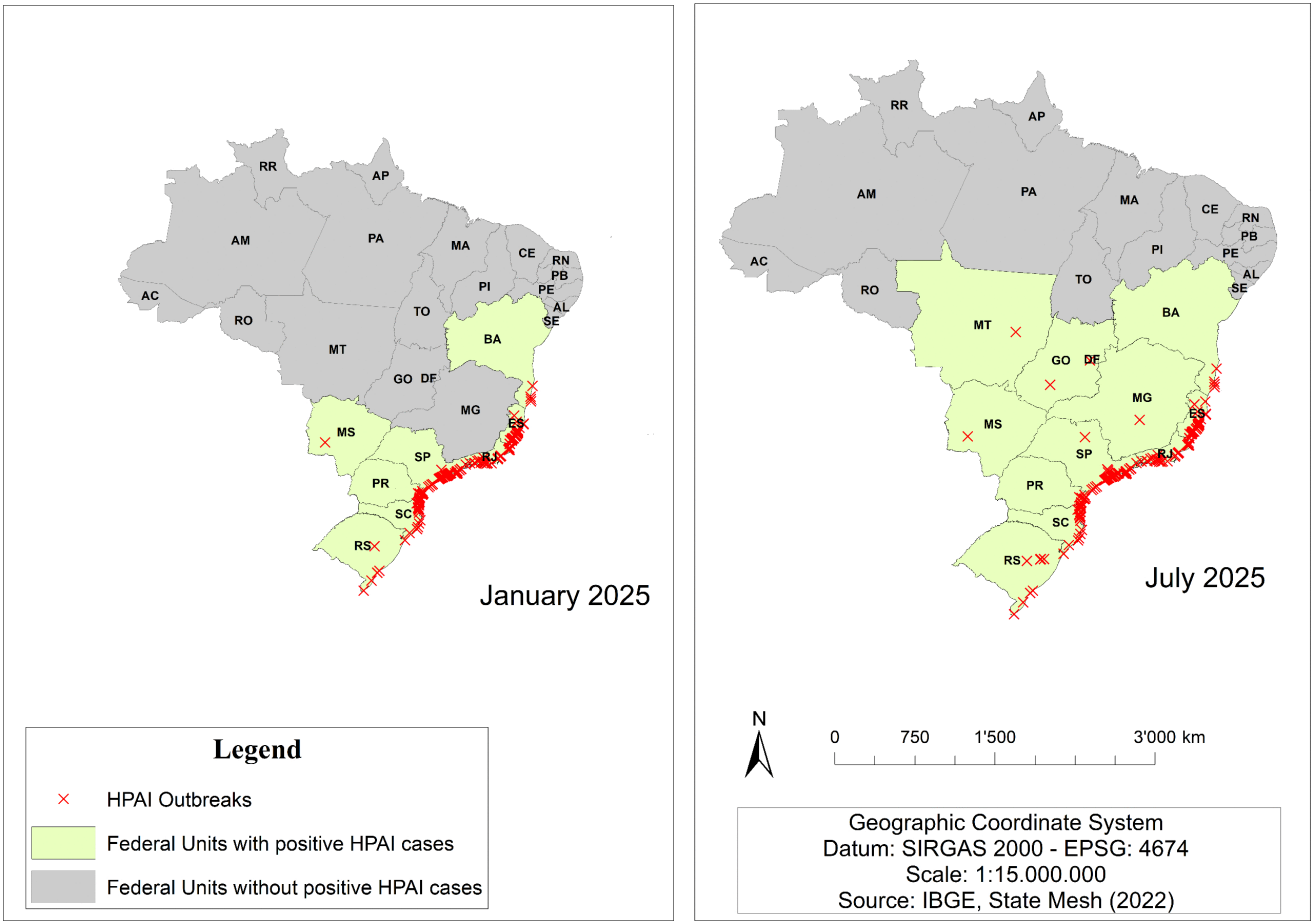
Spatiotemporal spread of HPAI outbreaks in Brazil (January – July 2025).

A comparison of the spatial distribution of HPAI outbreaks in Brazil between January and July 2025 (Fig 10) reveals the progression of the virus towards the interior of the country. Initially confined to eight federal units (Bahia, Espírito Santo, Rio de Janeiro, São Paulo, Paraná, Santa Catarina, Rio Grande do Sul, and Mato Grosso do Sul), the virus has since spread to include Minas Gerais, Goiás, Brasília, and Mato Grosso.

**Fig 11:**
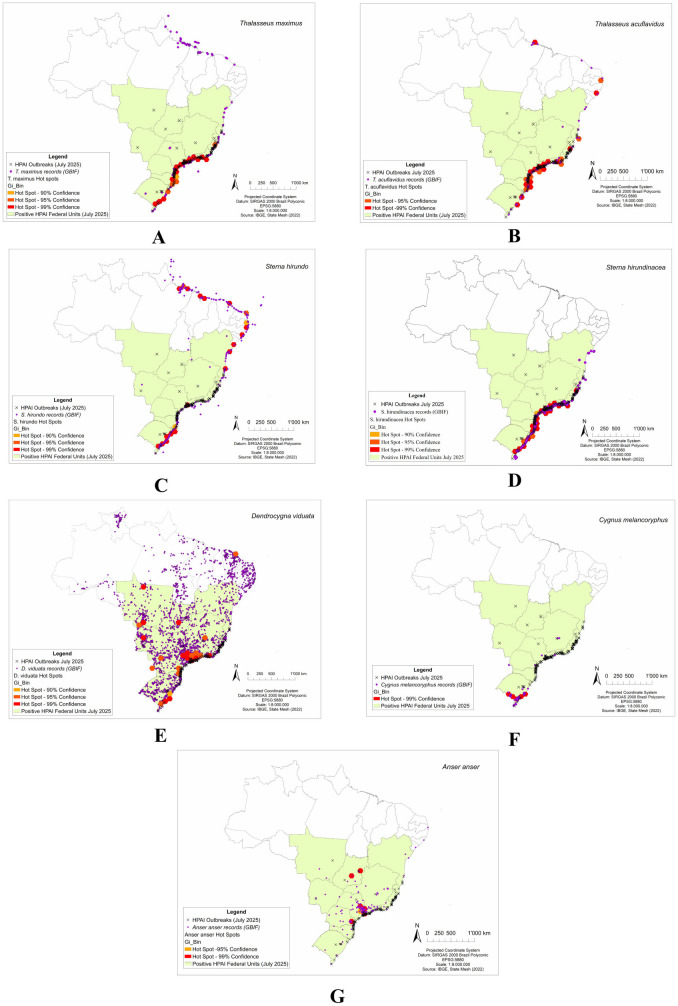
Species distribution, geographic hotspots, and HPAI outbreaks in Brazil (July 2025). Multi‑panel map (A–G) integrating GBIF occurrence records, spatial hotspot analysis (Getis‑Ord Gi*), and reported HPAI outbreaks in Brazil as of July 2025 for seven avian species previously identified as potential HPAI predictors. Panels show Thalasseus maximus (A), *Thalasseus acuflavidus* (B), *Sterna hirundo* (C), *Sterna hirundinacea* (D), *Dendrocygna viduata* (E), *Cygnus melancoryphus* (F), and *Anser anser* (G). Hotspots at 90–99% confidence are shown for each species. Figure credit: Brazilian Institute of Geography and Statistics – IBGE, State Mesh (2022).

Seven thematic maps were generated for Brazil by integrating the data layers described in the methodology section (Fig 11). The species included in the analysis were *T. acuflavidus*, *T. maximus*, *S. hirundo*, *S. hirundinacea*, *D. viduata*, *C. melancoryphus*, and *A. anser*.

Table 1 summarizes the number of records and the temporal range of GBIF observations used to construct each species’ distribution map and hotspots analysis. Records lacking geographic coordinates or presenting high spatial uncertainty were excluded from the dataset.

**Table 1:** Species Data for Hotspot Modeling.

Scientific name	Number of records (GBIF)	Range of record dates (GBIF)
*Thalasseus acuflavidus*	133	1865 - 2024
*Thalasseus maximus*	5240	1911 - 2024
*Sterna hirundinacea*	2352	1925 - 2024
*Sterna hirundo*	1494	1971 - 2024
*Dendrocygna viduata*	28131	1871- 2025
*Cygnus melancoryphus*	1026	1986 - 2024
*Anser anser*	712	1990 - 2025

Characteristics of the species occurrence data obtained from GBIF for modeling species distribution hotspots.

The bird species considered can be divided into two groups: seabirds with a coastal distribution (*T. maximus, T. acuflavidus, S. hirundinacea*, and *S. hirundo*) and waterfowl (*D. viduata, C. melancoryphus*, and *A. anser*), whose distribution includes both coastal and inland locations of the country.

When comparing the distribution of HPAI outbreaks with the geographic hotspots of the studied species, the greatest spatial overlap was observed with *T. acuflavidus* and *S. hirundinacea*. In contrast, *S. hirundo* and *T. maximus* exhibited broader geographic distributions that extend beyond the current range of the virus, including areas along the northern and eastern coasts where no outbreaks have been reported to date.

The distribution of HPAI outbreaks in Brazil remains primarily restricted to the coastal strip extending from Santa Vitória do Palmar (Rio Grande do Sul) to Porto Seguro (Bahia). However, sporadic inland cases have also been reported in locations such as Mateus Leme (Minas Gerais), Brasília (Federal District), Campinápolis (Mato Grosso), Santo Antônio da Barra (Goiás), Jaboticabal (São Paulo), Bonito (Mato Grosso do Sul), Rio Pardo, Montenegro, and Sapucaia do Sul (all in Rio Grande do Sul).

The coincidence between the hotspots of waterfowl species, particularly that of *D. viduata*, and the occurrence of new HPAI cases in the interior states of Brazil between January and July 2025 is interesting. The particularity of these species’ geographical distribution, which includes both coastal and inland locations, allows them to encounter marine birds that are hosts of the virus and become potential dispersers towards the interior.

Composite map illustrating the change in geographic distribution of highly pathogenic avian influenza (HPAI) outbreaks in Brazil between January and July 2025. Federal units are color‑coded to indicate the presence or absence of positive HPAI cases, while X markers denote specific outbreak locations. By July 2025, the geographic distribution of HPAI‑positive federal units had expanded or shifted, reflecting the spread of the virus over the six‑month period. Federal units without positive cases represent regions that remained free of detected HPAI outbreaks as of July 2025. Figure credit: Brazilian Institute of Geography and Statistics – IBGE, State Mesh (2022).

Occurrence records of *Thalasseus maximus* were concentrated along the southeastern and southern coasts. Hotspots at 90–99% confidence overlap partially with HPAI‑positive federal units and outbreak locations, particularly in São Paulo, Paraná, and Santa Catarina.

*Thalasseus acuflavidus* showed a strong spatial association with HPAI outbreaks. Hotspots at 95–99% confidence align closely with affected coastal areas, reinforcing its role as a key predictor (see Fig 6).

*Sterna hirundo* records were widely distributed along the coast, with 90–99% confidence hotspots in several HPAI‑positive states, especially in the Southeast and South.

*Sterna hirundinacea* occurrence was more restricted to southern Brazil. Hotspots at 90–99% confidence show moderate overlap with positive federal units.

*Dendrocygna viduata* occurred mainly in inland wetlands. Hotspots (90–99% confidence) appear in the South and parts of the Southeast, with some correspondence to HPAI‑positive areas.

*Cygnus melancoryphus* occurrence records were limited to southernmost Brazil. A 99% confidence hotspot is detected but shows less overlap with HPAI outbreaks given the species’ restricted range.

*Anser anser* occurrence records were sparse and localized. Hotspots at 95–99% confidence are small and scattered, with limited spatial coincidence with HPAI outbreaks.

## Discussion

The results of this study provide robust evidence for an association between the occurrence of highly pathogenic avian influenza (HPAI) outbreaks in Brazil and the presence of specific seabird and waterfowl species. By integrating official suspect records (DB1), statistical modelling, and geospatial analyses, we identified biologically and ecologically meaningful patterns with direct implications for epidemiological surveillance. This integrative framework not only advances the characterization of species that may serve as indicators of outbreak risk but also adds predictive quantification to the current understanding of coastal incursions. Our findings of exceptionally high positivity in some species like *Thalasseus/Sterna* and the latitudinal coastal gradient (Rio Grande do Sul→Bahia) align with the early incursion narrative proposed by De Araújo et. al and Rivetti et. al**. [**29,30], as well as with reports of impacts on marine mammals [29,31]. Importantly, the magnitude of the odds ratios in our model (e.g., *T. acuflavidus* OR ≈ 81) provides a highly actionable predictive dimension that complements these previous studies, which did not incorporate such quantitative risk estimates.

The remarkably high prevalence observed in *T. maximus* (94.5%) and *T. acuflavidus* (72.3%) represents compelling evidence for the role of seabirds in the epidemiology of HPAI in Brazil. Similarly, the associations detected in *S. hirundo* and *S. hirundinacea* reinforce the role of terns as hosts and dispersal agents.

Furthermore, *T. maximus*, *T. acuflavidus*, and *S. hirundinacea* are considered partially migratory species in Brazil, which strengthens their status as reservoirs and dispersers of the virus. They possess populations that reside and reproduce permanently in the country, forming dense coastal colonies in states such as Rio Grande do Sul (*T. maximus*), Espírito Santo (*T. acuflavidus*), and Santa Catarina (*S. hirundinacea*) [20]. These conditions facilitate viral transmission and amplification.

This aligns with international reports documenting the involvement of these species in coastal HPAI outbreaks in South America and beyond [1,32,33]. Collectively, these findings highlight seabird colonies as critical targets for systematic surveillance and underscore their potential use as early warning sentinels for novel incursions of HPAI along the Brazilian coastline.

In addition to seabirds, significant associations were observed for Anatidae species, particularly *C. melancoryphus* and *D. viduata*. Ducks, swans, and geese are established historic reservoirs of avian influenza viruses [34,35], with wild geese and swans being key facilitators of host jumps from wild to domestic populations [1].

Our results corroborate this evidence within the Brazilian context. The inclusion of these species as predictors in our models confirms that wetlands and continental water bodies, which serve as critical resting sites and ecological interfaces between wild bird groups, constitute high-risk environments for viral persistence and circulation. Consequently, identifying Anatidae as potential reservoirs and dispersers underscores the urgent need to strengthen surveillance systems focused on strategic aquatic habitats throughout Brazil.

The geographic distribution of HPAI outbreaks in Brazil showed a pronounced concentration along the coastal strip from Rio Grande do Sul to Bahia, with only sporadic incursions inland. This pattern suggests that the initial introduction of the virus was mediated by seabirds, consistent with the findings of Rivetti et al. [30], who, through a phylogeographic analysis of clade 2.3.4.4b A/H5N1 isolates, proposed a north-to-south dispersal route across the Americas via Central America and the Andes, entering Brazil through the southern coast and subsequently spreading northeastward. However, given that most infected species recorded in Brazil were seabirds, it appears unlikely that these species crossed the Andes to connect Pacific and Atlantic dispersal routes. A more plausible explanation lies in the role of coastal species such as *S. hirundinacea*, whose continuous distribution extends from the Peruvian coast to Tierra del Fuego in the Pacific and from Argentinian Patagonia to Brazil in the Atlantic [18], thereby facilitating a predominant south-to-north dispersal pathway along the Atlantic coast. Inland outbreaks likely represent secondary processes of spread, including long-distance migratory movements, indirect contact at continental wetlands, or under-detection of early cases. Moreover, the marked heterogeneity in outbreak proportions across municipalities, with positivity rates reaching 81.3% in those with ≥10 records, highlights sampling bias concentrated around confirmed outbreak sites, which constrains the generalizability of these results.

Comparative model analyses demonstrated that species composition constitutes a strong and highly informative predictor of HPAI outbreaks, with high discriminative performance (AUC 0.85–0.97). Notably, the spatial GAM eliminated residual autocorrelation and improved explanatory power, ensuring less biased estimates and greater inferential robustness. However, the persistence of minimal significant spatial autocorrelation in the GAM residuals suggests that even after explicitly modeling broad-scale spatial trends, there may be fine-scale spatial processes or very localized drivers of HPAI that are not captured by our current set of predictors. This could include factors such as hyper-local environmental conditions, unmeasured social networks, or data limitations at the municipal level. The significant reduction in spatial autocorrelation from the logit to the GAM model provides strong evidence that our spatial approach was necessary and correct, and the remaining spatial autocorrelation offers a crucial direction for future research.

Integrating biological predictors with spatial structure provides a powerful approach for developing early warning systems and prioritizing high-risk areas and species for targeted surveillance. Following the first HPAI outbreak in Brazil’s commercial poultry sector, surveillance priorities must pivot from coastal nodes and seabird populations to inland waterfowl and their migratory corridors. This strategic realignment will require the precise identification of key wetlands essential for foraging and roosting. Once validated in other settings, these methodologies could be scaled to a regional framework throughout Latin America and potentially to a continental level, offering a replicable and strategically valuable tool for addressing transboundary risk scenarios.

This study is not without limitations. Sampling was conditioned by official reporting of suspected cases, introducing bias toward municipalities with higher detection capacity. Species occurrence records were derived from heterogeneous sources, often with spatial and temporal gaps. Furthermore, the absence of key environmental covariates—such as wetland distribution, ocean currents, or poultry production density—likely constrained model explanatory power. Nevertheless, the methodological strengths, including cross-validation and control of spatial autocorrelation, provide confidence in the robustness of our findings.

To enhance future analyses and improve regional applicability, it is essential to integrate additional variables capturing the complexity of transmission dynamics. These include: (i) distribution and extent of wetlands and inland water bodies; (ii) intensity and seasonality of migratory flyways; (iii) density and location of backyard and commercial poultry systems; (iv) climatic drivers and ocean anomalies (e.g., El Niño) affecting migration and viral persistence; and (v) socio-economic variables related to biosecurity practices and local poultry markets. Incorporating these dimensions will allow the development of more accurate and generalizable models, strengthening their utility for large-scale prevention and preparedness strategies.

## Conclusions

This study provides evidence that specific seabird and waterfowl species are strongly associated with the spatial patterns of HPAI occurrence in Brazil. By integrating official surveillance records, biodiversity data, and spatial modeling, this work highlights the value of combining ecological and epidemiological information to identify species, locations, and interfaces that may deserve closer attention within risk-based surveillance systems. Rather than providing definitive evidence of transmission pathways, the models offer a practical framework for detecting epidemiological signals that can support preparedness and early warning efforts.

The findings can be directly used to inform guidance and policy in both the immediate and medium- to long-term. In the immediate term, surveillance authorities could use the species and areas identified in this study to prioritize targeted sampling, mortality event investigation, and rapid reporting in seabird colonies, wetlands, coastal municipalities, migratory bird concentration sites, and poultry–wildlife interfaces. These results may also help guide field teams in deciding where to intensify carcass searches, environmental monitoring, communication with poultry producers, and biosecurity advisories for commercial and backyard poultry systems located near high-risk ecological interfaces. In the medium and long term, the methodological framework could support the development of risk-based surveillance plans, allocation of diagnostic and field resources, integration of wildlife and poultry health data, and construction of regional early warning systems for HPAI in Brazil and other Latin American countries.

However, the risk patterns identified in this study should be interpreted as temporally conditioned signals, not as fixed maps of future risk. The species involved in HPAI detections, the relative contribution of migratory and resident birds, viral introduction routes, and areas of greatest concern may change between outbreak years and migratory seasons. Therefore, future applications of this framework should repeat the analysis using shorter and regularly updated analytical windows, such as annual periods, migratory seasons, or outbreak-specific phases. This would allow animal health authorities to assess whether the associations observed here remain stable over time or whether new species, regions, or ecological interfaces emerge as surveillance priorities. In this way, the framework could evolve into a dynamic decision-support tool capable of informing sampling strategies, field investigations, biosecurity guidance, and early warning actions in near real time.

This study also has several limitations that should be considered when interpreting the findings. First, the analysis relied on official surveillance data designed for early detection and outbreak response, rather than for systematic ecological sampling. Therefore, the high number of HPAI-positive records in some seabird species should not be interpreted automatically as evidence that these species are the main long-distance spreaders of the virus. In colonial seabirds, high positivity may also reflect greater susceptibility, higher mortality, easier detection during mortality events, coastal aggregation, or stronger surveillance attention in areas where carcasses are more likely to be found and reported. Thus, the species identified by the models should be understood as epidemiological indicators within the surveillance system, not necessarily as definitive reservoir species or primary drivers of viral dissemination.

Second, the models included broad ecological and migratory classifications, but they did not incorporate detailed species-specific movement traits such as migratory routes, stopover sites, flight distances, seasonal connectivity, foraging behavior, or the capacity of infected individuals to move while shedding viruses. This is particularly important because species may differ substantially in their epidemiological role: some may be highly susceptible and die close to the place of exposure, while others, such as certain ducks, geese, or other waterfowl, may move over longer distances and contribute more effectively to viral spread. In addition, the datasets analyzed did not include complete viral genomic information linked to each sampled species and location. Therefore, the spatial associations observed here should be interpreted as patterns of co-occurrence between surveillance detections, host species, and geography, rather than as direct evidence of transmission pathways, viral relatedness, or long-distance dispersal capacity.

Third, although cross-validation and spatial modeling strengthened the analytical framework, a key limitation was the inability to fully account for all spatial autocorrelation, as indicated by the significant Moran’s I observed in the GAM residuals. This suggests that some fine-scale spatial structure remained unexplained by the variables included in the models. Such residual spatial dependence may reflect unmeasured ecological, environmental, production-related, or surveillance-related factors, including wetland distribution, inland water bodies, poultry density, backyard poultry distribution, biosecurity practices, migratory connectivity, oceanographic conditions, climatic anomalies, or local poultry movements. Future studies should incorporate these covariates and explore alternative modeling frameworks, such as Geographically Weighted Regression or Bayesian hierarchical models with conditional autoregressive priors, to better account for spatial dependence at different scales.

Overall, if validated and periodically updated in broader settings, this framework could support a regional early warning system for Latin America and contribute to a continental strategy to anticipate and mitigate the risk of HPAI. Strengthening surveillance in seabird colonies, wetlands, coastal areas, migratory bird concentration sites, and poultry–wildlife interfaces, together with the integration of host movement data, viral genomics, environmental drivers, poultry production data, and socio-economic information, will be critical to improve the actionable value of predictive models for transboundary animal health and One Health preparedness.

## Supporting information

S1 File Collection of suspected HPAI samples in Brazil (DB1). This file contains the complete dataset of suspected and confirmed HPAI events in Brazil, updated to July 7, 2025. Variables include federal unit, municipality, occurrence ID, date of veterinary service, main species recorded, farming or management type, case status, and date of conclusive diagnosis.
(XLSX)

S2 File Georeferenced HPAI outbreak data in Brazil (DB2). This file contains the complementary georeferenced register of confirmed HPAI and Newcastle disease events in Brazil. Variables include disease diagnosis, case status, farming type, location, scientific name of the species, main-species indicator, investigation number (linked to DB1), report date, and geographic coordinates.
(XLSX)

S3 File Spatial analysis dataset for optimized logistic regression model. This file contains the complete dataset used to evaluate the spatial independence of residuals from the optimized logistic regression model. It includes geographic coordinates for each municipality (obtained from the centroid of its administrative polygon, EPSG:4326) used to construct the k-nearest neighbors (kNN, Haversine) spatial weights matrix.
(XLSX)
