# Supplementary material for: Spatial association of seabirds and aquatic birds with highly pathogenic avian influenza (H5N1) outbreaks in Brazil: A nationwide ecological and statistical modelling approach
Source: PLoS One. 2026 Jul 1;21(7):e0350505. doi: 10.1371/journal.pone.0350505 (PMC13322515; doi:10.1371/journal.pone.0350505)
Supplement: Attachment — Submitted filename: Response to Reviewers.docx [file pone.0350505.s005.docx]

Dear Editor and Reviewers,

We thank the Editor and the reviewers for their thoughtful comments and constructive suggestions. We have carefully addressed all the points raised. Below we provide a point-by-point response. All changes in the manuscript have been highlighted (or are described in detail where applicable).

1. Journal Requirements

1.1 PLOS ONE style requirements

Answer: The adjustments included in the templates have been made.

1.2 Code sharing

Answer: We did not generate our own code; we used available code for the analyses performed, as described in the manuscript.

1.3 Ethics statement location

Answer: The ethics statement appears only in the Methods section.

1.4 Data Availability Statement and minimal data set

Answer: Thank you for your observation. At this time, not all supplementary information files are included with the current submission. However, we confirm that we will upload all relevant raw data and supporting information files required to replicate the study results at the stage of manuscript resubmission. We are currently organizing the complete minimal data set — including the values behind the reported means, standard deviations, graph data, and any extracted points from images — and will upload them as Supporting Information files.

1.5 Data availability on acceptance

Answer: We confirm that all data required to replicate the study findings will be shared as Supporting Information files. No public repository deposition is planned, as the data will be fully accessible through the article's supplementary materials. We will ensure that the minimal data set is included in the Supporting Information files at the time of resubmission.

1.6 Funding information mismatch

Answer: Thank you for noticing this discrepancy. We will make the respective corrections when we resubmit the manuscript. We will ensure that the grant information provided in both the 'Funding Information' and 'Financial Disclosure' sections matches, and that the correct grant numbers are included.

1.7 Authorship list in manuscript file

Answer: We will amend the authorship list in the manuscript file to include the following authors when we resubmit the article:

Byron Abdel Hernandez-Ortiz

Angela Maria Arcila-Cardona

Camila Michele Appolinario

Thiago Araujo Dos Santos

1.8 Authorship list in submission system

Answer: We will amend the manuscript submission data via "Edit Submission" to include Byron A. Hernández-Ortiz, Thiago Araujo dos Santos, Camila M. Appolinário, and Angela M. Arcila-Cardona when we resubmit the article.

1.9 Copyrighted images in Figure 11

Answer: Figures 11A–G were modified by removing the images of the birds. Regarding the maps: they are of our own creation, generated using the base cartography of Brazil freely available on the IBGE website (https://www.ibge.gov.br/). The Brazilian Institute of Geography and Statistics (IBGE) is Brazil's official statistical and geographic data provider, and it provides free, open-access cartographic data.

1.10 Copyrighted maps in Figures 8, 10, and 11

Answer: Figures 8, 10, and 11 are maps of our own creation, generated using the base cartography of Brazil freely available on the IBGE website (https://www.ibge.gov.br/). IBGE provides free, open-access cartographic data. No proprietary or copyrighted map data (e.g., from Google) were used.

1.11 Citation of previously published works requested by reviewers

Answer: We have carefully reviewed the comments received from the reviewers. None of the reviewers requested the citation of any specific previously published works. Therefore, we have not added any additional citations beyond those already present in the manuscript.

2. Responses to Reviewers' Comments

Reviewer #1

General comment: This manuscript outlines an interesting and timely study; however, the manuscript is a bit hard to follow due to the highly technical nature of the modeling work. Can this be explained more clearly for a broader audience? The figures are relevant and useful but could be improved with the addition of more informative figure legends. The conclusions are valid for the most part but there could be more discussion about the limitations.

2.1.1 Technical language and accessibility

Answer: We thank the reviewer for this important observation. We revised the methodological description to make the data sources and surveillance context clearer for a broader audience. In particular:

We simplified the explanation of the official surveillance system.

We clarified the distinction between passive and active risk-based surveillance.

We explicitly explained why the dataset should not be interpreted as a random sample of all wild bird species or geographic regions.

We revised the description of DB1 and DB2 to improve readability.

We added a clearer explanation of potential sampling imbalance and how this limitation was considered when interpreting species-level and spatial associations.

2.1.2 Limitations discussion

Answer: We expanded the limitations section to more clearly discuss the constraints of using official surveillance data for ecological risk modeling. Specifically, we now address:

Potential reporting and sampling biases.

The non-random nature of the surveillance data.

The limitations of inferring causality from ecological associations.

Heterogeneity of species occurrence records.

Possible temporal mismatches between occurrence and outbreak data.

The absence of important environmental, ecological, production-related, and socio-economic covariates.

We also clarified that the species-level associations should be interpreted as epidemiological signals within the available surveillance data, rather than as direct evidence of infection prevalence or causal transmission roles.

2.1.3 Species susceptibility, migratory patterns, and viral genetics

Answer: The reviewer raises excellent points. We acknowledge the following:

Susceptibility vs. dispersal: A high number of HPAI-positive samples from some seabird species may indeed indicate high susceptibility and possibly limited viral dispersal. We have added a discussion of this nuance in the limitations and interpretation sections.

Migratory patterns and behavior: We did not include migratory patterns, behavior, or flight distances explicitly in the models. This is now stated as a limitation and a future research direction.

Genetic profiles: The available data did not include systematic genetic characterization of the isolated HPAI viruses. We now note that including viral genetic information would be valuable to distinguish between species that are primarily affected and those that may act as long-distance reservoirs (e.g., geese and ducks). This has been added to the discussion as a recommendation for future studies.

We have also searched the literature for available information on susceptibility, displacement distances, and migratory patterns of the prioritized species (e.g., *Thalasseus acuflavidus*, *Sterna hirundo*, *Anser anser*, *Cygnus melancoryphus*) and have incorporated this where relevant.

2.1.4 Improve figures and figure legends

Answer: We have improved the figures and added more descriptive legends for Figures 1–11. In particular:

Figures 1, 2, 3, 5, and 6 were improved in quality and clarity.

Figure 4 was converted into a map as suggested.

Figure 11 was modified to remove copyrighted bird images; the maps are original using IBGE open-access cartography.

All figure legends now include more detailed explanations of the data, statistical methods, and how to interpret the visual elements.

Reviewer #2

General comment: This is a very interesting and valuable study, methodologically sound. Only a few comments.

2.2.1 Policy and guidance implications (conclusion section)

Answer: We thank the reviewer for this important suggestion. We revised the conclusion section to explicitly clarify how the findings can be used for guidance and policy. We added a new paragraph explaining:

Immediate applicability: Risk-based surveillance, targeted sampling, investigation of mortality events, intensified monitoring in seabird colonies, wetlands, coastal municipalities, migratory bird concentration sites, and poultry–wildlife interfaces, as well as support for biosecurity advisories in nearby poultry systems.

Medium- and long-term implications: Use of this framework for surveillance planning, resource allocation, integration of wildlife and poultry health data, and development of regional early warning systems for HPAI in Brazil and Latin America.

2.2.2 Dynamic risk and temporal updates

Answer: We agree that HPAI risk is dynamic. We revised the conclusion section to clarify that the proposed framework should not be interpreted as a static risk map, but as a dynamic tool requiring periodic updating. We now explicitly state that future applications should repeat the analysis using shorter and updated time windows, such as annual periods, migratory seasons, or outbreak-specific phases. This would allow surveillance authorities to evaluate whether the species and areas identified remain stable over time or whether new host groups, regions, or ecological interfaces emerge as priorities.

2.2.3 Detailed description of sampling, data collection, and reporting

Answer: We revised the Methods section to provide a more detailed explanation:

Notification and sampling procedures during wild bird mortality events are now described.

Unusual mortality events were notified to the Official Veterinary Service by environmental agencies, producers, veterinarians, technical personnel, or citizens, and handled by the local veterinary unit.

Field investigations included site inspection, interviews, verification of abnormal mortality or compatible clinical signs, epidemiological investigation, sample collection, and registration in e-Sisbravet.

Suspected events meeting official criteria for probable respiratory and neurological syndrome were sampled under passive surveillance protocols.

Samples (tracheal/cloacal swabs, tissues from necropsies) were tested for influenza A by RT-qPCR; positive/inconclusive samples were further tested for H5, H5 clade 2.3.4.4, and N1, with partial hemagglutinin sequencing in selected cases.

We also note that the system did not change substantially during the study period, and mass mortality events followed the same investigation protocol.

2.2.4 Sampling bias towards certain species

Answer: We agree that a species-level sampling bias is possible. We have clarified this limitation in the Methods and Discussion sections:

The surveillance data are not a random or fully representative sample of all wild bird species in Brazil.

Passive surveillance was based on notifications of suspected events or unusual mortality, prioritizing aquatic and migratory birds (Anseriformes and Charadriiformes) and relying partly on environmental monitoring networks (beach monitoring, conservation units).

Species that are large, colonial, coastal, water-associated, abundant, or easier to detect/identify are more likely to be reported and sampled.

Observer familiarity may also contribute, but the overrepresentation of some taxa appears driven mainly by the risk-based design and detectability during mortality events.

We interpreted species-level associations as surveillance-derived risk signals, not as direct estimates of infection prevalence.

2.2.5 Typographical corrections

Answer: We have corrected the following:

Line 63: Changed "World Health Organization (WHO)" to "World Organisation for Animal Health (WOAH)" as suggested.

Title: Corrected "modelling" spelling.

Line 337: Changed "Anser answer" to "Anser anser".

"Significative" changed to "significant with p < 0.001" or similar as appropriate.

2.2.6 Figure quality

Answer:

Figures 1, 2, and 5 were difficult to read in the PDF but are clear in the downloadable TIFF files. We have ensured that the resubmitted figures have improved resolution and readability.

Figure 2: We have added text labels to the first bars for consistency.

Figure 4: We have converted this into a map as suggested, since readers may not be familiar with Brazilian federal units.

3. Additional changes

All changes made in the manuscript have been tracked or are clearly described in this response letter. We believe that the revised manuscript is substantially improved and hope it is now acceptable for publication in PLOS ONE.

Thank you again for your time and constructive feedback.

Sincerely,

The authors
