# Supplementary material for: Spatial association of seabirds and aquatic birds with highly pathogenic avian influenza (H5N1) outbreaks in Brazil: A nationwide ecological and statistical modelling approach
Source: PLoS One. 2026 Jul 1;21(7):e0350505. doi: 10.1371/journal.pone.0350505 (PMC13322515; doi:10.1371/journal.pone.0350505)
Supplement: Peer review history

# Peer review: Decision Letter 0

**DOI:** 10.1371/journal.pone.0350505.r001

**Authors:** Clement Adebajo Meseko

20 Mar 2026

PONE-D-25-65727

Spatial association of seabirds and aquatic birds with highly pathogenic avian influenza (H5N1) outbreaks in Brazil: A nationwide ecological and statistical modeling approach

PLOS One

Dear Dr. Hernandez-Ortiz,

Thank you for submitting your manuscript to PLOS ONE. After careful consideration, we feel that it has merit but does not fully meet PLOS ONE’s publication criteria as it currently stands. Therefore, we invite you to submit a revised version of the manuscript that addresses the points raised during the review process.

Please submit your revised manuscript by May 04 2026 11:59PM. If you will need more time than this to complete your revisions, please reply to this message or contact the journal office at plosone@plos.org. When you're ready to submit your revision, log on to https://www.editorialmanager.com/pone/ and select the 'Submissions Needing Revision' folder to locate your manuscript file.

Please include the following items when submitting your revised manuscript:

- A letter that responds to each point raised by the academic editor and reviewer(s). You should upload this letter as a separate file labeled 'Response to Reviewers'.
- A marked-up copy of your manuscript that highlights changes made to the original version. You should upload this as a separate file labeled 'Revised Manuscript with Track Changes'.
- An unmarked version of your revised paper without tracked changes. You should upload this as a separate file labeled 'Manuscript'.

If you would like to make changes to your financial disclosure, please include your updated statement in your cover letter. Guidelines for resubmitting your figure files are available below the reviewer comments at the end of this letter.

If applicable, we recommend that you deposit your laboratory protocols in protocols.io to enhance the reproducibility of your results. Protocols.io assigns your protocol its own identifier (DOI) so that it can be cited independently in the future. For instructions see: https://journals.plos.org/plosone/s/submission-guidelines#loc-laboratory-protocols. Additionally, PLOS ONE offers an option for publishing peer-reviewed Lab Protocol articles, which describe protocols hosted on protocols.io. Read more information on sharing protocols at https://plos.org/protocols?utm_medium=editorial-email&utm_source=authorletters&utm_campaign=protocols.

We look forward to receiving your revised manuscript.

Kind regards,

Clement Adebajo Meseko, DVM, PhD

Academic Editor

PLOS One

Journal Requirements:

When submitting your revision, we need you to address these additional requirements.

1. Please ensure that your manuscript meets PLOS ONE's style requirements, including those for file naming. The PLOS ONE style templates can be found at

https://journals.plos.org/plosone/s/file?id=wjVg/PLOSOne_formatting_sample_main_body.pdf and https://journals.plos.org/plosone/s/file?id=ba62/PLOSOne_formatting_sample_title_authors_affiliations.pdf

2. Please note that PLOS One has specific guidelines on code sharing for submissions in which author-generated code underpins the findings in the manuscript. In these cases, we expect all author-generated code to be made available without restrictions upon publication of the work. Please review our guidelines at https://journals.plos.org/plosone/s/materials-and-software-sharing#loc-sharing-code and ensure that your code is shared in a way that follows best practice and facilitates reproducibility and reuse.

3. Your ethics statement should only appear in the Methods section of your manuscript. If your ethics statement is written in any section besides the Methods, please delete it from any other section.

4. We note that your Data Availability Statement is currently as follows: All relevant data are within the manuscript and its Supporting Information files.

Please confirm at this time whether or not your submission contains all raw data required to replicate the results of your study. Authors must share the “minimal data set” for their submission. PLOS defines the minimal data set to consist of the data required to replicate all study findings reported in the article, as well as related metadata and methods (https://journals.plos.org/plosone/s/data-availability#loc-minimal-data-set-definition).

For example, authors should submit the following data:

- The values behind the means, standard deviations and other measures reported;

- The values used to build graphs;

- The points extracted from images for analysis.

Authors do not need to submit their entire data set if only a portion of the data was used in the reported study.

If your submission does not contain these data, please either upload them as Supporting Information files or deposit them to a stable, public repository and provide us with the relevant URLs, DOIs, or accession numbers. For a list of recommended repositories, please see https://journals.plos.org/plosone/s/recommended-repositories.

If there are ethical or legal restrictions on sharing a de-identified data set, please explain them in detail (e.g., data contain potentially sensitive information, data are owned by a third-party organization, etc.) and who has imposed them (e.g., an ethics committee). Please also provide contact information for a data access committee, ethics committee, or other institutional body to which data requests may be sent. If data are owned by a third party, please indicate how others may request data access.

5. When completing the data availability statement of the submission form, you indicated that you will make your data available on acceptance. We strongly recommend all authors decide on a data sharing plan before acceptance, as the process can be lengthy and hold up publication timelines. Please note that, though access restrictions are acceptable now, your entire data will need to be made freely accessible if your manuscript is accepted for publication. This policy applies to all data except where public deposition would breach compliance with the protocol approved by your research ethics board. If you are unable to adhere to our open data policy, please kindly revise your statement to explain your reasoning and we will seek the editor's input on an exemption. Please be assured that, once you have provided your new statement, the assessment of your exemption will not hold up the peer review process.

6. We note that the grant information you provided in the ‘Funding Information’ and ‘Financial Disclosure’ sections do not match.

When you resubmit, please ensure that you provide the correct grant numbers for the awards you received for your study in the ‘Funding Information’ section.

7. Please amend your authorship list in your manuscript file to include author Byron Abdel Hernandez-Ortiz, Angela Maria Arcila-Cardona, Camila Michele Appolinario, Thiago Araujo DOs Santos.

8. Please amend the manuscript submission data (via Edit Submission) to include author Byron A. Hernández-Ortiz, Thiago Araujo dos Santos, Camila M. Appolinário, Angela M. Arcila-Cardona.

9. We note that Figure 11 in your submission contain copyrighted images. All PLOS content is published under the Creative Commons Attribution License (CC BY 4.0), which means that the manuscript, images, and Supporting Information files will be freely available online, and any third party is permitted to access, download, copy, distribute, and use these materials in any way, even commercially, with proper attribution. For more information, see our copyright guidelines: http://journals.plos.org/plosone/s/licenses-and-copyright.

We require you to either (1) present written permission from the copyright holder to publish these figures specifically under the CC BY 4.0 license, or (2) remove the figures from your submission:

a. You may seek permission from the original copyright holder of Figure 11 to publish the content specifically under the CC BY 4.0 license.

We recommend that you contact the original copyright holder with the Content Permission Form (http://journals.plos.org/plosone/s/file?id=7c09/content-permission-form.pdf) and the following text:

“I request permission for the open-access journal PLOS ONE to publish XXX under the Creative Commons Attribution License (CCAL) CC BY 4.0 (http://creativecommons.org/licenses/by/4.0/). Please be aware that this license allows unrestricted use and distribution, even commercially, by third parties. Please reply and provide explicit written permission to publish XXX under a CC BY license and complete the attached form.”

Please upload the completed Content Permission Form or other proof of granted permissions as an "Other" file with your submission.

In the figure caption of the copyrighted figure, please include the following text: “Reprinted from [ref] under a CC BY license, with permission from [name of publisher], original copyright [original copyright year].”

b. If you are unable to obtain permission from the original copyright holder to publish these figures under the CC BY 4.0 license or if the copyright holder’s requirements are incompatible with the CC BY 4.0 license, please either i) remove the figure or ii) supply a replacement figure that complies with the CC BY 4.0 license. Please check copyright information on all replacement figures and update the figure caption with source information. If applicable, please specify in the figure caption text when a figure is similar but not identical to the original image and is therefore for illustrative purposes only.

10. We note that Figure(s) 8, 10, 11 in your submission contain [map/satellite] images which may be copyrighted. All PLOS content is published under the Creative Commons Attribution License (CC BY 4.0), which means that the manuscript, images, and Supporting Information files will be freely available online, and any third party is permitted to access, download, copy, distribute, and use these materials in any way, even commercially, with proper attribution. For these reasons, we cannot publish previously copyrighted maps or satellite images created using proprietary data, such as Google software (Google Maps, Street View, and Earth). For more information, see our copyright guidelines: http://journals.plos.org/plosone/s/licenses-and-copyright.

We require you to either (1) present written permission from the copyright holder to publish these figures specifically under the CC BY 4.0 license, or (2) remove the figures from your submission:

a. You may seek permission from the original copyright holder of Figure(s) 8, 10, 11 to publish the content specifically under the CC BY 4.0 license.

We recommend that you contact the original copyright holder with the Content Permission Form (http://journals.plos.org/plosone/s/file?id=7c09/content-permission-form.pdf) and the following text:

“I request permission for the open-access journal PLOS ONE to publish XXX under the Creative Commons Attribution License (CCAL) CC BY 4.0 (http://creativecommons.org/licenses/by/4.0/). Please be aware that this license allows unrestricted use and distribution, even commercially, by third parties. Please reply and provide explicit written permission to publish XXX under a CC BY license and complete the attached form.”

Please upload the completed Content Permission Form or other proof of granted permissions as an "Other" file with your submission.

In the figure caption of the copyrighted figure, please include the following text: “Reprinted from [ref] under a CC BY license, with permission from [name of publisher], original copyright [original copyright year].”

b. If you are unable to obtain permission from the original copyright holder to publish these figures under the CC BY 4.0 license or if the copyright holder’s requirements are incompatible with the CC BY 4.0 license, please either i) remove the figure or ii) supply a replacement figure that complies with the CC BY 4.0 license. Please check copyright information on all replacement figures and update the figure caption with source information. If applicable, please specify in the figure caption text when a figure is similar but not identical to the original image and is therefore for illustrative purposes only.

The following resources for replacing copyrighted map figures may be helpful:

USGS National Map Viewer (public domain): http://viewer.nationalmap.gov/viewer/

The Gateway to Astronaut Photography of Earth (public domain): http://eol.jsc.nasa.gov/sseop/clickmap/

Maps at the CIA (public domain): https://www.cia.gov/library/publications/the-world-factbook/index.html and https://www.cia.gov/library/publications/cia-maps-publications/index.html

NASA Earth Observatory (public domain): http://earthobservatory.nasa.gov/

Landsat: http://landsat.visibleearth.nasa.gov/

USGS EROS (Earth Resources Observatory and Science (EROS) Center) (public domain): http://eros.usgs.gov/#

Natural Earth (public domain): http://www.naturalearthdata.com/

11. If the reviewer comments include a recommendation to cite specific previously published works, please review and evaluate these publications to determine whether they are relevant and should be cited. There is no requirement to cite these works unless the editor has indicated otherwise.

[Note: HTML markup is below. Please do not edit.]

Reviewers' comments:

Reviewer's Responses to Questions

**Comments to the Author**

1. Is the manuscript technically sound, and do the data support the conclusions?

The manuscript must describe a technically sound piece of scientific research with data that supports the conclusions. Experiments must have been conducted rigorously, with appropriate controls, replication, and sample sizes. The conclusions must be drawn appropriately based on the data presented.

Reviewer #1: Partly

Reviewer #2: Yes

**********

2. Has the statistical analysis been performed appropriately and rigorously?

Reviewer #1: I Don't Know

Reviewer #2: Yes

**********

3. Have the authors made all data underlying the findings in their manuscript fully available?

The PLOS Data policy requires authors to make all data underlying the findings described in their manuscript fully available without restriction, with rare exception (please refer to the Data Availability Statement in the manuscript PDF file). The data should be provided as part of the manuscript or its supporting information, or deposited to a public repository. For example, in addition to summary statistics, the data points behind means, medians and variance measures should be available. If there are restrictions on publicly sharing data—e.g. participant privacy or use of data from a third party—those must be specified.

Reviewer #1: Yes

Reviewer #2: Yes

**********

4. Is the manuscript presented in an intelligible fashion and written in standard English?

PLOS ONE does not copyedit accepted manuscripts, so the language in submitted articles must be clear, correct, and unambiguous. Any typographical or grammatical errors should be corrected at revision, so please note any specific errors here.

Reviewer #1: Yes

Reviewer #2: Yes

**********

5. Review Comments to the Author

Please use the space provided to explain your answers to the questions above. You may also include additional comments for the author, including concerns about dual publication, research ethics, or publication ethics. (Please upload your review as an attachment if it exceeds 20,000 characters)

Reviewer #1: This manuscript outlines an interesting and timely study however, the manuscript is a bit hard to follow due to the highly technical nature of the modeling work. Can this be explained more clearly for a broader audience ? The figures are relevant and useful but could be improved with the addition of more informative figure legends. The conclusions are valid for the most part but there could be more discussion about the limitations of this sort of study. For example, the high number of HPAI positive samples from some species of sea bird might indicate that these species are highly susceptible and might not spread the virus far. Were the migratory patterns, behavior and flight distances of each species included in the models used ? Did the data available include the genetic profiles of the HPAI viruses isolated ? for example, we might like to know if the latter were the same in all species mentioned in the manuscript or if there was variation ? The latter is important when considering 'risk' and for generating models about which species are most impacted vs reservoir species spreading HPAI long distances such as (potentially) geese and ducks.

Reviewer #2: The manuscript “Spatial association of seabirds and aquatic birds with highly pathogenic avian influenza (H5N1) outbreaks in Brazil: A nationwide ecological and statistical modeling approach” is a very interesting and valuable study to assess the distribution of animal (bird) species and HPAI in Brazil. It is very well conducted, methodologically sound and provides a lot of very useful information that can also be used for risk assessment approaches in the One Health context.

There are only a few comments that the authors could consider:

I miss in the conclusion section a paragraph how the results of this study can be directly utilised for guidance and policy on an immediate and medium/long term.

It is mentioned that the framework can be expanded across the South American Region or used in other countries to develop risk mapping frameworks, which is a reasonable proposal. The current analysis uses quite a large year range and susceptible bird species or infected migratory birds bringing the disease to specific areas might change over time or even between migratory years. How will you address this and is it planned to repeat the analysis using shorter/updated time periods to support risk assessments?

It is briefly mentioned in the discussion section, but a more detailed explanation in the introduction or methods section would help to improve the understanding about the underlying sampling, data collection and reporting. Please describe how the sampling is performed (deaths, active/passive surveillance, specialised teams/authorities or everybody,…) and how the data reporting and collection to the two repositories is happening to provide a better understanding of the data as well as the limitations and possible biases. Has the system how suspected animals have been sampled and/or reported changed during the period included in your study? How were sampling and reporting conducted during mass mortality events at sites? Please provide some more details.

Could there be a sampling bias towards specific species e.g. for which the species name might be better known, or which were better known by the people collecting the data?

Line 63: “a total of 19 countries and territories in the Americas reported 4,713 H5N1 avian influenza outbreaks to the World Health Organization (WHO),” - please check if you mean WHO or WOAH

Please check some typos in the manuscript:

Check typo in the title (modelling)

Line 337: Anser answer – check and revise to anser anser – likely an autocorrection error

Significative? Significant with P<…? Please check and correct wording.

Figures:

The figures 1,2 and 5 are very difficult to understand or read due to the low quality in the pdf (better in the downloadable tiff).

Fig 2, please be consistent and add text also to first bars

Fig 4, would be nicer to have as a map – the reader might not be familiar with the federal units in Brazil

**********

6. PLOS authors have the option to publish the peer review history of their article (what does this mean?). If published, this will include your full peer review and any attached files.

If you choose “no”, your identity will remain anonymous but your review may still be made public.

**Do you want your identity to be public for this peer review?** For information about this choice, including consent withdrawal, please see our Privacy Policy.

Reviewer #1: No

Reviewer #2: No

**********

[NOTE: If reviewer comments were submitted as an attachment file, they will be attached to this email and accessible via the submission site. Please log into your account, locate the manuscript record, and check for the action link "View Attachments". If this link does not appear, there are no attachment files.]

To ensure your figures meet our technical requirements, please review our figure guidelines: https://journals.plos.org/plosone/s/figures

You may also use PLOS’s free figure tool, NAAS, to help you prepare publication quality figures: https://journals.plos.org/plosone/s/figures#loc-tools-for-figure-preparation.

NAAS will assess whether your figures meet our technical requirements by comparing each figure against our figure specifications.

# Author comment: Author response to Decision Letter 1

**DOI:** 10.1371/journal.pone.0350505.r002

5 May 2026

1. Journal Requirements

1.1 PLOS ONE style requirements

Answer: The adjustments included in the templates have been made.

1.2 Code sharing

Answer: We did not generate our own code; we used available code for the analyses performed, as described in the manuscript.

1.3 Ethics statement location

Answer: The ethics statement appears only in the Methods section.

1.4 Data Availability Statement and minimal data set

Answer: Thank you for your observation. At this time, not all supplementary information files are included with the current submission. However, we confirm that we will upload all relevant raw data and supporting information files required to replicate the study results at the stage of manuscript resubmission. We are currently organizing the complete minimal data set — including the values behind the reported means, standard deviations, graph data, and any extracted points from images — and will upload them as Supporting Information files.

1.5 Data availability on acceptance

Answer: We confirm that all data required to replicate the study findings will be shared as Supporting Information files. No public repository deposition is planned, as the data will be fully accessible through the article's supplementary materials. We will ensure that the minimal data set is included in the Supporting Information files at the time of resubmission.

1.6 Funding information mismatch

Answer: Thank you for noticing this discrepancy. We will make the respective corrections when we resubmit the manuscript. We will ensure that the grant information provided in both the 'Funding Information' and 'Financial Disclosure' sections matches, and that the correct grant numbers are included.

1.7 Authorship list in manuscript file

Answer: We will amend the authorship list in the manuscript file to include the following authors when we resubmit the article:

Byron Abdel Hernandez-Ortiz

Angela Maria Arcila-Cardona

Camila Michele Appolinario

Thiago Araujo Dos Santos

1.8 Authorship list in submission system

Answer: We will amend the manuscript submission data via "Edit Submission" to include Byron A. Hernández-Ortiz, Thiago Araujo dos Santos, Camila M. Appolinário, and Angela M. Arcila-Cardona when we resubmit the article.

1.9 Copyrighted images in Figure 11

Answer: Figures 11A–G were modified by removing the images of the birds. Regarding the maps: they are of our own creation, generated using the base cartography of Brazil freely available on the IBGE website (https://www.ibge.gov.br/). The Brazilian Institute of Geography and Statistics (IBGE) is Brazil's official statistical and geographic data provider, and it provides free, open-access cartographic data.

1.10 Copyrighted maps in Figures 8, 10, and 11

Answer: Figures 8, 10, and 11 are maps of our own creation, generated using the base cartography of Brazil freely available on the IBGE website (https://www.ibge.gov.br/). IBGE provides free, open-access cartographic data. No proprietary or copyrighted map data (e.g., from Google) were used.

1.11 Citation of previously published works requested by reviewers

Answer: We have carefully reviewed the comments received from the reviewers. None of the reviewers requested the citation of any specific previously published works. Therefore, we have not added any additional citations beyond those already present in the manuscript.

2. Responses to Reviewers' Comments

Reviewer #1

General comment: This manuscript outlines an interesting and timely study; however, the manuscript is a bit hard to follow due to the highly technical nature of the modeling work. Can this be explained more clearly for a broader audience? The figures are relevant and useful but could be improved with the addition of more informative figure legends. The conclusions are valid for the most part but there could be more discussion about the limitations.

2.1.1 Technical language and accessibility

Answer: We thank the reviewer for this important observation. We revised the methodological description to make the data sources and surveillance context clearer for a broader audience. In particular:

We simplified the explanation of the official surveillance system.

We clarified the distinction between passive and active risk-based surveillance.

We explicitly explained why the dataset should not be interpreted as a random sample of all wild bird species or geographic regions.

We revised the description of DB1 and DB2 to improve readability.

We added a clearer explanation of potential sampling imbalance and how this limitation was considered when interpreting species-level and spatial associations.

2.1.2 Limitations discussion

Answer: We expanded the limitations section to more clearly discuss the constraints of using official surveillance data for ecological risk modeling. Specifically, we now address:

Potential reporting and sampling biases.

The non-random nature of the surveillance data.

The limitations of inferring causality from ecological associations.

Heterogeneity of species occurrence records.

Possible temporal mismatches between occurrence and outbreak data.

The absence of important environmental, ecological, production-related, and socio-economic covariates.

We also clarified that the species-level associations should be interpreted as epidemiological signals within the available surveillance data, rather than as direct evidence of infection prevalence or causal transmission roles.

2.1.3 Species susceptibility, migratory patterns, and viral genetics

Answer: The reviewer raises excellent points. We acknowledge the following:

Susceptibility vs. dispersal: A high number of HPAI-positive samples from some seabird species may indeed indicate high susceptibility and possibly limited viral dispersal. We have added a discussion of this nuance in the limitations and interpretation sections.

Migratory patterns and behavior: We did not include migratory patterns, behavior, or flight distances explicitly in the models. This is now stated as a limitation and a future research direction.

Genetic profiles: The available data did not include systematic genetic characterization of the isolated HPAI viruses. We now note that including viral genetic information would be valuable to distinguish between species that are primarily affected and those that may act as long-distance reservoirs (e.g., geese and ducks). This has been added to the discussion as a recommendation for future studies.

We have also searched the literature for available information on susceptibility, displacement distances, and migratory patterns of the prioritized species (e.g., Thalasseus acuflavidus, Sterna hirundo, Anser anser, Cygnus melancoryphus) and have incorporated this where relevant.

2.1.4 Improve figures and figure legends

Answer: We have improved the figures and added more descriptive legends for Figures 1–11. In particular:

Figures 1, 2, 3, 5, and 6 were improved in quality and clarity.

Figure 4 was converted into a map as suggested.

Figure 11 was modified to remove copyrighted bird images; the maps are original using IBGE open-access cartography.

All figure legends now include more detailed explanations of the data, statistical methods, and how to interpret the visual elements.

Reviewer #2

General comment: This is a very interesting and valuable study, methodologically sound. Only a few comments.

2.2.1 Policy and guidance implications (conclusion section)

Answer: We thank the reviewer for this important suggestion. We revised the conclusion section to explicitly clarify how the findings can be used for guidance and policy. We added a new paragraph explaining:

Immediate applicability: Risk-based surveillance, targeted sampling, investigation of mortality events, intensified monitoring in seabird colonies, wetlands, coastal municipalities, migratory bird concentration sites, and poultry–wildlife interfaces, as well as support for biosecurity advisories in nearby poultry systems.

Medium- and long-term implications: Use of this framework for surveillance planning, resource allocation, integration of wildlife and poultry health data, and development of regional early warning systems for HPAI in Brazil and Latin America.

2.2.2 Dynamic risk and temporal updates

Answer: We agree that HPAI risk is dynamic. We revised the conclusion section to clarify that the proposed framework should not be interpreted as a static risk map, but as a dynamic tool requiring periodic updating. We now explicitly state that future applications should repeat the analysis using shorter and updated time windows, such as annual periods, migratory seasons, or outbreak-specific phases. This would allow surveillance authorities to evaluate whether the species and areas identified remain stable over time or whether new host groups, regions, or ecological interfaces emerge as priorities.

2.2.3 Detailed description of sampling, data collection, and reporting

Answer: We revised the Methods section to provide a more detailed explanation:

Notification and sampling procedures during wild bird mortality events are now described.

Unusual mortality events were notified to the Official Veterinary Service by environmental agencies, producers, veterinarians, technical personnel, or citizens, and handled by the local veterinary unit.

Field investigations included site inspection, interviews, verification of abnormal mortality or compatible clinical signs, epidemiological investigation, sample collection, and registration in e-Sisbravet.

Suspected events meeting official criteria for probable respiratory and neurological syndrome were sampled under passive surveillance protocols.

Samples (tracheal/cloacal swabs, tissues from necropsies) were tested for influenza A by RT-qPCR; positive/inconclusive samples were further tested for H5, H5 clade 2.3.4.4, and N1, with partial hemagglutinin sequencing in selected cases.

We also note that the system did not change substantially during the study period, and mass mortality events followed the same investigation protocol.

2.2.4 Sampling bias towards certain species

Answer: We agree that a species-level sampling bias is possible. We have clarified this limitation in the Methods and Discussion sections:

The surveillance data are not a random or fully representative sample of all wild bird species in Brazil.

Passive surveillance was based on notifications of suspected events or unusual mortality, prioritizing aquatic and migratory birds (Anseriformes and Charadriiformes) and relying partly on environmental monitoring networks (beach monitoring, conservation units).

Species that are large, colonial, coastal, water-associated, abundant, or easier to detect/identify are more likely to be reported and sampled.

Observer familiarity may also contribute, but the overrepresentation of some taxa appears driven mainly by the risk-based design and detectability during mortality events.

We interpreted species-level associations as surveillance-derived risk signals, not as direct estimates of infection prevalence.

2.2.5 Typographical corrections

Answer: We have corrected the following:

Line 63: Changed "World Health Organization (WHO)" to "World Organisation for Animal Health (WOAH)" as suggested.

Title: Corrected "modelling" spelling.

Line 337: Changed "Anser answer" to "Anser anser".

"Significative" changed to "significant with p < 0.001" or similar as appropriate.

2.2.6 Figure quality

Answer:

Figures 1, 2, and 5 were difficult to read in the PDF but are clear in the downloadable TIFF files. We have ensured that the resubmitted figures have improved resolution and readability.

Figure 2: We have added text labels to the first bars for consistency.

Figure 4: We have converted this into a map as suggested, since readers may not be familiar with Brazilian federal units.

Attachment Submitted filename: Response to Reviewers.docx

# Peer review: Decision Letter 1

**DOI:** 10.1371/journal.pone.0350505.r003

**Authors:** Clement Adebajo Meseko

15 May 2026

<p>Spatial association of seabirds and aquatic birds with highly pathogenic avian influenza (H5N1) outbreaks in Brazil: A nationwide ecological and statistical modelling approach

PONE-D-25-65727R1

Dear Dr. Hernandez-Ortiz,

We’re pleased to inform you that your manuscript has been judged scientifically suitable for publication and will be formally accepted for publication once it meets all outstanding technical requirements.

Within one week, you’ll receive an e-mail detailing the required amendments. When these have been addressed, you’ll receive a formal acceptance letter and your manuscript will be scheduled for publication.

An invoice will be generated when your article is formally accepted. Please note, if your institution has a publishing partnership with PLOS and your article meets the relevant criteria, all or part of your publication costs will be covered. Please make sure your user information is up-to-date by logging into Editorial Manager at Editorial Manager® and clicking the ‘Update My Information' link at the top of the page. For questions related to billing, please contact billing support.

If your institution or institutions have a press office, please notify them about your upcoming paper to help maximize its impact. If they’ll be preparing press materials, please inform our press team as soon as possible -- no later than 48 hours after receiving the formal acceptance. Your manuscript will remain under strict press embargo until 2 pm Eastern Time on the date of publication. For more information, please contact onepress@plos.org.

Kind regards,

Clement Adebajo Meseko, DVM, PhD

Academic Editor

PLOS One

Additional Editor Comments (optional):

Reviewers' comments:

Reviewer's Responses to Questions

**Comments to the Author**

1. If the authors have adequately addressed your comments raised in a previous round of review and you feel that this manuscript is now acceptable for publication, you may indicate that here to bypass the “Comments to the Author” section, enter your conflict of interest statement in the “Confidential to Editor” section, and submit your "Accept" recommendation.

Reviewer #1: All comments have been addressed

Reviewer #2: All comments have been addressed

**********

2. Is the manuscript technically sound, and do the data support the conclusions?

The manuscript must describe a technically sound piece of scientific research with data that supports the conclusions. Experiments must have been conducted rigorously, with appropriate controls, replication, and sample sizes. The conclusions must be drawn appropriately based on the data presented.

Reviewer #1: Yes

Reviewer #2: Yes

**********

3. Has the statistical analysis been performed appropriately and rigorously?

Reviewer #1: Yes

Reviewer #2: Yes

**********

4. Have the authors made all data underlying the findings in their manuscript fully available?

The PLOS Data policy requires authors to make all data underlying the findings described in their manuscript fully available without restriction, with rare exception (please refer to the Data Availability Statement in the manuscript PDF file). The data should be provided as part of the manuscript or its supporting information, or deposited to a public repository. For example, in addition to summary statistics, the data points behind means, medians and variance measures should be available. If there are restrictions on publicly sharing data—e.g. participant privacy or use of data from a third party—those must be specified.

Reviewer #1: Yes

Reviewer #2: Yes

**********

5. Is the manuscript presented in an intelligible fashion and written in standard English?

PLOS ONE does not copyedit accepted manuscripts, so the language in submitted articles must be clear, correct, and unambiguous. Any typographical or grammatical errors should be corrected at revision, so please note any specific errors here.

Reviewer #1: Yes

Reviewer #2: Yes

**********

6. Review Comments to the Author

Please use the space provided to explain your answers to the questions above. You may also include additional comments for the author, including concerns about dual publication, research ethics, or publication ethics. (Please upload your review as an attachment if it exceeds 20,000 characters)

Reviewer #1: (No Response)

Reviewer #2: The revised version reads much clearer and all comments have been addressed. The results section has been rewritten and the policy issues covered.

**********

7. PLOS authors have the option to publish the peer review history of their article (what does this mean?). If published, this will include your full peer review and any attached files.

If you choose “no”, your identity will remain anonymous but your review may still be made public.

**Do you want your identity to be public for this peer review?** For information about this choice, including consent withdrawal, please see our Privacy Policy.

Reviewer #1: **Yes:**Dr Susan Catherine Cork

Reviewer #2: No

**********

# Editor report: Acceptance letter

**DOI:** 10.1371/journal.pone.0350505.r004

**Authors:** Clement Adebajo Meseko

PONE-D-25-65727R1

PLOS One

Dear Dr. Hernandez-Ortiz,

I'm pleased to inform you that your manuscript has been deemed suitable for publication in PLOS One. Congratulations! Your manuscript is now being handed over to our production team.

At this stage, our production department will prepare your paper for publication. This includes ensuring the following:

* All references, tables, and figures are properly cited

* All relevant supporting information is included in the manuscript submission,

* There are no issues that prevent the paper from being properly typeset

You will receive further instructions from the production team, including instructions on how to review your proof when it is ready. Please keep in mind that we are working through a large volume of accepted articles, so please give us a few days to review your paper and let you know the next and final steps.

Lastly, if your institution or institutions have a press office, please let them know about your upcoming paper now to help maximize its impact. If they'll be preparing press materials, please inform our press team within the next 48 hours. Your manuscript will remain under strict press embargo until 2 pm Eastern Time on the date of publication. For more information, please contact onepress@plos.org.

You will receive an invoice from PLOS for your publication fee after your manuscript has reached the completed accept phase. If you receive an email requesting payment before acceptance or for any other service, this may be a phishing scheme. Learn how to identify phishing emails and protect your accounts at https://explore.plos.org/phishing.

If we can help with anything else, please email us at customercare@plos.org.

Thank you for submitting your work to PLOS ONE and supporting open access.

Kind regards,

PLOS ONE Editorial Office Staff

on behalf of

Dr. Clement Adebajo Meseko

Academic Editor

PLOS One
